# The penta-EF-hand protein Pef1 of *Candida albicans* functions at sites of membrane perturbation to support polarized growth and membrane integrity

**DOI:** 10.1093/g3journal/jkag075

**Published:** 2026-04-01

**Authors:** Martin Weichert, Marcel René Schumann, Ulrike Brandt, Cameron Bedford, Alexandra C Brand, André Fleißner

**Affiliations:** Institut für Genetik, Technische Universität Braunschweig, Braunschweig 38106, Germany; Institut für Genetik, Technische Universität Braunschweig, Braunschweig 38106, Germany; Institut für Genetik, Technische Universität Braunschweig, Braunschweig 38106, Germany; Medical Research Council Centre for Medical Mycology (MRC CMM) at the University of Exeter, Exeter EX4 4QD, United Kingdom; Medical Research Council Centre for Medical Mycology (MRC CMM) at the University of Exeter, Exeter EX4 4QD, United Kingdom; Institut für Genetik, Technische Universität Braunschweig, Braunschweig 38106, Germany

**Keywords:** *Candida albicans*, cell membrane integrity, membrane stress, membrane repair, polyene, saponin, cell polarity, polarized growth, PEF-hand protein, Pef1, calcium, calcineurin, pathogenicity

## Abstract

The fungal plasma membrane is the target of fungicidal compounds, such as polyenes and saponins, that directly interact with fungus-specific ergosterol to cause deleterious membrane disruption. To counter membrane attack, diverse eukaryotic cells employ Ca^2+^-binding penta-EF (PEF)-hand proteins, including the human ortholog, ALG-2, to maintain membrane integrity. *Candida albicans* is a major fungal pathogen in humans, where increasing resistance to current antifungal drugs that target the plasma membrane is of serious concern. Combinatorial treatments that additionally compromise the plasma membrane offer a way forward, but our mechanistic understanding of how fungi respond to direct membrane disruption remains limited. Here, we investigated the PEF-hand ortholog, Pef1, in this polymorphic species. GFP-tagged Pef1 localized at sites of polarized growth in yeast and hyphal cells of *C. albicans*. On treatment of hyphae with the polyene drug, amphotericin B, or the saponin tomatine, GFP-Pef1 became distributed as punctate spots at the membrane. In a similar manner, loss of calcineurin A (Cna1), but not of its transcription factor, Crz1, caused this punctate localization pattern of GFP-Pef1. While deletion of *PEF1* slightly impaired yeast cell growth rate, filamentation was not affected. Strikingly, *pef1*Δ hyphae could not maintain plasma membrane integrity in serum, as also seen in the *cna1*Δ mutant, and exhibited attenuated virulence in an insect larvae infection model. Together, these observations suggest that Pef1 localizes to sites of membrane perturbation to maintain cell integrity, including sites of dynamic polarized growth, septum formation, and fungicide-induced membrane disruption.

## Introduction

Maintaining and promoting the integrity and function of cell membranes is vital for all eukaryotic organisms, including fungi. Although fungal cells are surrounded by dynamic cell walls that provide major protection from mechanical, chemical, or osmotic insults (Gow et al. 2017), the underlying plasma membrane can still be exposed to various forms of stress that pose a major threat to cellular integrity and survival. Fungal lipid bilayers contain ergosterol, a sterol that is fungus-specific and therefore an attractive target in the search for compounds that directly interact with it (Perfect, 2017; Puumala et al. 2024). Ergosterol is crucial for maintaining both the barrier function of the plasma membrane and the functionality of membrane-associated and transmembrane proteins that support cell wall structure, nutrition, cell polarity, and stress signaling pathways of fungal cells (Athanasopoulos et al. 2019; Lanze et al. 2020). Ergosterol-binding antifungal metabolites from the polyene and saponin classes, which are unrelated fungicides naturally produced by bacteria and plants, quickly disrupt fungal cell membrane integrity via ergosterol extraction or deleterious membrane-pore formation (Carolus et al. 2020; Augustin et al. 2011). Because of their fungicidal activity, nystatin and amphotericin B are used as polyene drugs in the treatment of infectious diseases caused by human-pathogenic fungi, including *Aspergillus fumigatus* and *Candida albicans* (Köhler et al. 2017; Chandrasekar 2011). Infections of plants caused by phytopathogenic fungi such as *Botrytis cinerea* are often followed by the release of antifungal saponins, eg α-tomatine from tomato plants, sparking interest in understanding how saponins could be exploited to combat fungal infections and to boost plant defenses (You and van Kan 2021; Trdá et al. 2019). To combat pathogenic fungi in clinical and agricultural settings, the azole class of antifungals is widely employed to block ergosterol biosynthesis, but their indirect and relatively slow effect on fungal membranes is mostly fungistatic and can select for antifungal resistance, threatening the applicability of azoles (Perfect 2017). Although fungicides, as well as membrane-disrupting antifungal immune responses in humans, animals, and plants, are highly relevant and efficient in directly targeting fungal membranes (Harpf et al. 2020; Struyfs et al. 2021), our current knowledge about the molecular basis of the membrane stress response in fungi during attack and defense remains limited.

The general membrane stress response in fungi involves the activation of important signaling pathways, including the well-studied Ca^2+^/calcineurin-dependent transcriptional response (Yadav and Heitman 2023; Park et al. 2019). However, this signaling pathway does not explain how fungi immediately respond to direct and acute forms of membrane disruption. In light of this, our previous study in the model fungus, *Neurospora crassa*, identified a role for the penta-EF (PEF)-hand protein, PEF1, in mediating a quick response to deleterious membrane disruption that promotes cellular survival (Schumann et al. 2019). Pef1 belongs to a class of cytosolic eukaryotic proteins that feature 5 highly conserved Ca^2+^-binding helix-loop-helix motifs (Maki et al. 2002). In *N. crassa*, the PEF1 protein facilitates membrane integrity during plasma membrane merger during somatic cell fusion. Strikingly, PEF1 is recruited to the ergosterol-rich polarized hyphal tips during the exposure to nystatin or tomatine, and this protein directly contributes to the tolerance of the saponin (Schumann et al. 2019). This protective role against tomatine was also demonstrated in *B. cinerea*, in which the orthologous PEF-hand protein contributes to the virulence of environmental isolates of this phytopathogenic species (You et al. 2024).

Research on the membrane-protective role of PEF-hand proteins in the context of host–pathogen interactions has also been shown in human cells, which can be attacked by numerous microbial membrane-pore forming toxins or mechanical forces that disrupt host cell membranes (Moyes et al. 2016; Jimenez and Perez 2017; Westman et al. 2018, 2019, 2022; Lapaquette et al. 2022). In their study, Westman *et al.* demonstrated that the human PEF-hand protein, ALG-2, maintains epithelial cell integrity during membrane attack by invasive hyphae of *C. albicans* that secrete the pore-forming peptide toxin, candidalysin (Westman et al. 2022). ALG-2 is a preformed Ca^2+^ sensor protein that mediates a membrane repair response by initiating assembly of the ESCRT protein complex, which seals membrane wounds by removing the damaged membrane area (Jimenez et al. 2014; Scheffer et al. 2014; La Cour et al. 2018). In human and fungal cells, the membrane recruitment of PEF-hand proteins is dependent on external Ca^2+^ (Schumann et al. 2019; Scheffer et al. 2014), indicating that the increase in cytosolic Ca^2+^ levels after membrane wounding acts as a common localized signal to activate these membrane stress responses. However, other similarities and possible differences in the membrane repair mechanisms used by different types of eukaryotic cells, including diverse fungi, remain unknown.

In this study, we investigated the role of PEF-hand proteins in promoting fungal membrane integrity and virulence using *C. albicans* as a representative of a human–pathogen (Köhler et al. 2017). As part of the human microbiome, *C. albicans* can cause opportunistic infections, which remain challenging to combat given the morphological plasticity and metabolic adaptability of this species, and the occurrence of resistance to antifungal drugs (Noble et al. 2017; Kumamoto et al. 2020; Lee et al. 2021). During the commensal and pathogenic lifestyles of this polymorphic fungus, yeast and hyphal cells can encounter membrane stress caused by antifungal attack by immune cells (Harpf et al. 2020; Chen et al. 2023). In particular, the constitutively polarized invasive hyphae of *C. albicans* require that membrane integrity is tightly controlled at their ergosterol-rich cell tips, which elongate by incorporating new membrane lipids and proteins through vesicle exocytosis (Douglas and Konopka 2016; Serrano et al. 2023). In the model organism, *Saccharomyces cerevisiae,* the Pef1 ortholog plays a role in the polarized growth phase of dividing yeast cells (Vernarecci et al. 2007). Here, we investigated Pef1 function during polarized growth of *C. albicans* yeast cells and filaments, in the presence and absence of membrane-perturbing compounds. The study revealed that Pef1 localizes to sites of membrane perturbation, including sites of membrane re-organization during polarized growth and septum formation, and at sites of membrane damage caused by membrane-targeting drugs. Thus, the function of Pef1 in *C. albicans* aligns with the role of PEF-hand proteins in eukaryotic membrane repair, while carrying additional significance in this pathogen, in which the plasma membrane is considered a fungus-specific target for novel antifungal drug development.

## Results

### Pef1 is required for polarized growth and resistance to SDS and Ca^2+^-depletion in yeast cells of *C. albicans*

Using PEF1 from *N. crassa* as a query for a BLASTp search, we identified an uncharacterized PEF-hand protein in *C. albicans*, which we hence designate Pef1. As annotated in the *Candida* Genome Database (http://www.candidagenome.org/), the diploid genome of *C. albicans* contains 2 identical alleles of a 1,116-bp intronless gene (C2_08020C_A and C2_08020C_B), which encode the Pef1 protein with a length of 371 amino acids (aa). A BLASTp search with the full-length aa sequence of Pef1 from *C. albicans* as a query revealed that this predicted PEF-hand protein shares approximately 38 and 29% of total identity with the *N. crassa* PEF1 and *S. cerevisiae* Pef1 proteins, respectively (Vernarecci et al. 2007; Schumann et al. 2019). Moreover, Pef1 in *C. albicans* is ∼33% identical to the human cytosolic Ca^2+^ sensor protein, ALG-2, which initiates plasma membrane repair during mechanical and chemical forms of membrane disruption (Westman et al. 2022; Scheffer et al. 2014). Pef1 also shows ∼34% identity with peflin, another human PEF-hand protein that interacts with ALG-2 in the context of ER-to-Golgi transport (Sargeant et al. 2021). Functional predictions and aa alignments of these PEF-hand proteins indicated that the 5 helix-loop-helix motifs, which form the Ca^2+^-binding PEF-hand region, are conserved between the human and fungal orthologs (Supplementary Fig. 1a). In contrast to ALG-2, peflin and its fungal orthologs possess an additional N-terminal extension with predicted disordered regions that show relatively low sequence identity or similarity to each other, in contrast to the conserved PEF-hand domains (Supplementary Fig. 1b). Pef1 appears to be the only penta-EF-hand protein encoded in the *C. albicans* genome.

**Fig. 1.**
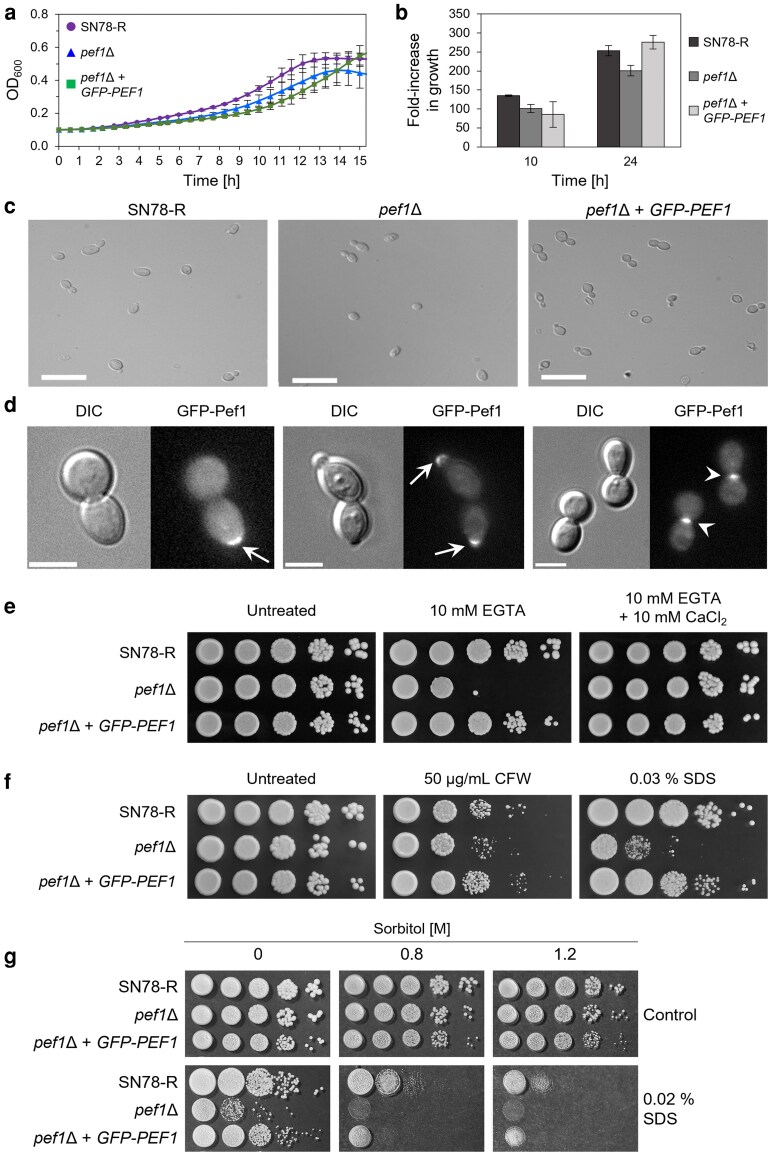
Pef1 supports polarized growth and is required for resistance to SDS and EGTA in *C. albicans* yeast cells. a) Growth of the control strain SN78-R (MW-Ca81), the *pef1*Δ mutant (MW-Ca27), and the *pef1*Δ strain complemented with *GFP-PEF1* (MW-Ca58) for 24 h at 30 °C in YPD broth in microtiter plates. The growth curves show the mean OD_600_ values with errors (standard deviation, Std Dev) represented as bars from 2 biological replicates. b) Measurement of growth of the strains from panel A at 37 °C in YPD broth in shaking flasks. The OD_600_ values were normalized for each strain and the relative fold-increase in growth was determined at the indicated time points. Bars = mean ± Std Dev from 2 biological replicates. c) Differential interference contrast (DIC) images of budding yeast cells from the strains described in panel A after 3 h of incubation at 30 °C in YPD broth. Scale bars: 20 μm. d) DIC and fluorescence microscopy images of yeast cells from the GFP-Pef1 reporter strain (MW-Ca58) in synthetic defined (SD) medium supplemented with 5 mM Ca^2+^. GFP-Pef1 localizes at the cortex of the emerging daughter cells (arrows) and at the neck in between the dividing cells (arrowheads) during yeast cell budding. Scale bars: 5 μm. e) Colonies of the strains indicated in panel A were grown in the presence or absence of the Ca^2+^-chelator EGTA. Ten-fold serial dilutions of yeast cells were spotted onto YPD agar plates with the indicated supplements and incubated for 2 d at 37 °C. f) Growth of colonies as described in panel E on YPD agar plates with and without calcofluor white (CFW) or sodium dodecyl sulphate (SDS). Images of the colonies were captured after 2 d of incubation at 37 °C. g): Sorbitol does not protect the *pef1*Δ mutant from SDS. Growth of colonies from 10-fold serial dilutions of yeast cells of SN78-R (MW-Ca81), the *pef1*Δ mutant (MW-Ca27), and the complemented mutant (MW-Ca58) on YPD agar plates supplemented with SDS and/or sorbitol. The images of the colonies were captured after incubation for 2 d at 37 °C.

To functionally characterize Pef1 in *C. albicans*, we generated a gene deletion mutant (*pef1*Δ) and transformed this with *N*-terminally tagged GFP-PEF1 (*pef1*Δ+*GFP-PEF1*) as a re-integrant control strain (Supplementary Fig. 2). When grown in YPD broth, *pef1*Δ achieved a lower final OD_600_ value at 30 or 37 °C than the wild-type *PEF1* control strain, SN78-R (Fig. 1a and b). Although delayed in germination, the specific growth rate of the complemented *pef1*Δ + *GFP-PEF1* strain was similar to that of SN78-R after ∼3 h (Fig. 1a and b), suggesting that 2 functional copies of *PEF1* are required for normal germination. Microscopy showed that the cell morphology of the *pef1*Δ mutant when grown as yeast was the same as that of the control strain (Fig. 1c). Consistent with a proposed role of Pef1 in polarized growth in *S. cerevisiae* (Vernarecci et al. 2007), the GFP-Pef1 fusion protein in *C. albicans* yeast cells also localized at the bud tip during early growth before translocation to the bud neck during septum formation (Fig. 1d). Since the establishment and maintenance of cell polarity are Ca^2+^-dependent processes (Sakaguchi et al. 1997), we next tested whether loss of Pef1 affected growth under Ca^2+^-limited conditions. Compared to the control strain, deletion of *PEF1* resulted in reduced growth on YPD agar plates containing the Ca^2+^ chelator, EGTA. This was rescued by supplementation of the medium with Ca^2+^, or by complementation with *GFP-PEF1* (Fig. 1e). The *pef1*Δ mutant was only slightly more susceptible to the cell wall-disrupting agent, calcofluor white (CFW) than the control strains. In contrast, exposure to sodium dodecyl sulfate (SDS), a surfactant that primarily disrupts cell membrane integrity, strongly impaired the growth of the *pef1*Δ mutant (Fig. 1f). Since SDS also impacts the cell wall (Cao et al. 2020), we supplemented the medium with sorbitol, which osmotically stabilizes cells with cell wall defects (Klis et al. 2002). However, sorbitol did not reverse the specific growth defect of the *pef1*Δ mutant in the presence of SDS (Fig. 1g). Taken together, these results indicate that Pef1 in *C. albicans* yeast cells functions at sites undergoing plasma-membrane remodeling and plays a role in resistance to membrane disruption by SDS.

### GFP-Pef1 localizes at the hyphal tip and is required for plasma membrane integrity in extending apical compartments

**Fig. 2.**
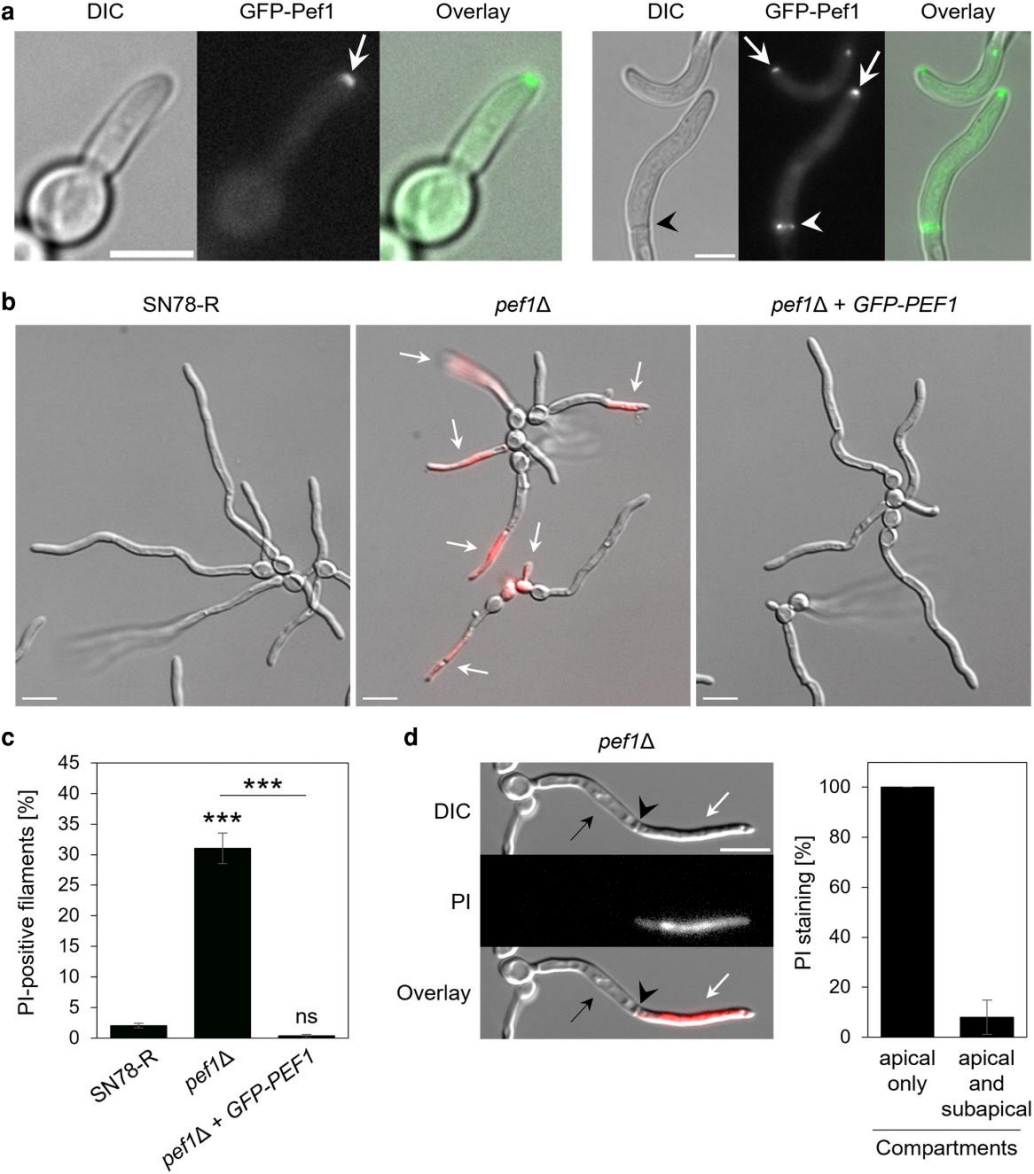
Pef1 localizes at the hyphal tip and facilitates membrane integrity of *C. albicans* hyphae. a) Incubation of the GFP-Pef1 reporter strain (MW-Ca58) in 20% fetal bovine serum (FBS) to induce the formation of filaments. Images were captured by fluorescence microscopy after 1 h (left panel) and 3 h (right panel) of incubation at 37 °C. GFP-Pef1 is localizing at the hyphal tip (arrows) and at a septum (black and white arrowheads). Scale bars: 5 μm. b): Overlay images (DIC and fluorescence) of filaments formed by SN78-R (MW-Ca81), the *pef1*Δ mutant (MW-Ca27), and the complemented strain (MW-Ca58) after 3 h of incubation at 37 °C in FBS. Staining with propidium iodide (PI) reveals accumulation of the red-fluorescent dye in the filaments of the *pef1*Δ mutant (arrows). Scale bars: 10 μm. c): Quantification of the percentage of filaments from the strains shows in panel B that stained positive with PI after 3 h of incubation at 37 °C in FBS. The black bars show the mean values with errors (Std Dev) from 3 independent experiments per strain. Statistically significant differences (***, *P* < 0.001; ns, not significant) were assessed by one-way ANOVA analysis with Tukey's correction for multiple comparisons. d): Quantification of PI staining patterns in septated PI-positive filaments of the *pef1*Δ mutant after 3–4 h of incubation at 37 °C in FBS. Left: DIC and fluorescent images of a representative PI-positive *pef1*Δ filament with one septum (arrowhead) separating an apical (white arrow) and subapical (back arrow) compartment. Scale bar: 10 μm. Right: Percentage of PI staining in apical and/or neighboring subapical hyphal compartments, with mean values and error bars (Std Dev) retrieved from a total of 52 septated hyphae in 4 independent experiments.

We next examined the localization of GFP-Pef1 during the filamentous growth of *C. albicans*. In 20% serum, GFP-Pef1 localized to hyphal tips and also to septa (Fig. 2a), consistent with a function for Pef1 at sites undergoing active membrane remodeling in yeast. Deletion of *pef1* did not affect the emergence or extension rate of hyphal filaments (Supplementary Fig. 3a and b). However, mutant hyphae were prone to showing signs of stress and damage indicated by the aberrant distribution and morphology of vacuoles. Strikingly, cell bursting was observed near the hyphal apex (Supplementary Fig. 3b and c). Staining of filaments with propidium iodide (PI), a red-fluorescent, DNA-binding dye that only enters cells with a compromised plasma membrane (Carmona-Gutierrez et al. 2018), indicated that the apical hyphal compartments of the *pef1*Δ hyphae were permeable to this compound (Fig. 2b). Approximately one third of the *pef1*Δ filaments were PI-positive (Fig. 2c), and this primarily occurred in the apical compartments, with subapical compartments remaining PI-negative (Fig. 2d). In contrast, hyphae of the *PEF1*-complemented strain were morphologically indistinguishable from the control strain and exhibited negative PI-staining (Fig. 2b and c). Treatment of *pef1*Δ hyphae with the lipophilic, red-fluorescent dye FM4-64 showed that ∼ 31% (58/188) internalized FM4-64 within 15 min compared to only ∼ 2% (2/96) of SN78-R hyphae, in which the dye predominantly labeled the cell periphery at this time point (Supplementary Fig. 3c). Overall, these observations suggest that the polarized localization of Pef1 is required to maintain integrity of the plasma membrane during normal hyphal growth of *C. albicans*.

The ability of *C. albicans* to form filaments in liquid media can be separated from its capacity to form hyphae that invade semisolid substrates (Sudbery 2011). Although hyphae of the *pef1*Δ mutant showed a defect in sustaining apical integrity in liquid (20%) serum (Fig. 2b), the filaments formed by colonies on solid (10%) serum agar showed no defect and were able to reach invasion depths similar to the control strains at both 30 °C or 37 °C (Supplementary Fig. 4a and b). Spider agar invasion at 30 °C or 37 °C was also not significantly affected by the deletion of *PEF1* (Supplementary Fig. 4c–e) incubation in liquid Spider medium did not significantly increase PI staining of the filaments of *pef1*Δ in contrast to the defect seen on exposure to liquid serum (Supplementary Fig. 5a and b). The observation that Pef1 is required for normal hyphal growth in 20% serum but not in 10% serum-agar or in solid or liquid Spider medium suggests that the *pef1*Δ mutant may be susceptible to Ca^2+^ stress at the higher calcium concentration in 20% FBS (0.7 mM Ca^2+^) but not in 10% FBS (0.35 mM Ca^2+^).

### The impaired hyphal integrity of the calcineurin mutant correlates with an altered tip localization of Pef1

**Fig. 3.**
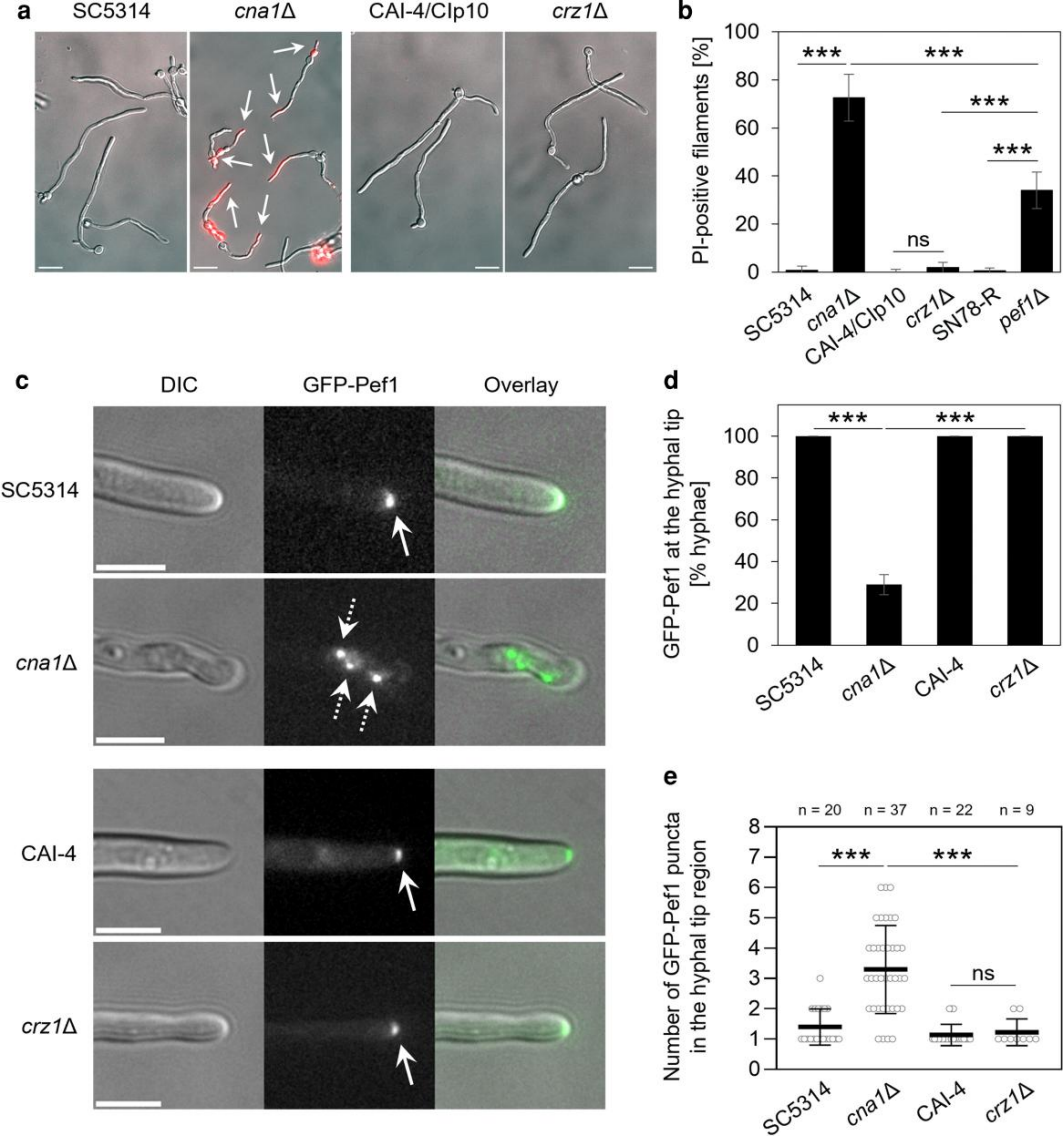
Loss of calcineurin impairs hyphal integrity and alters the distribution pattern of Pef1. a): Overlay images (DIC and red fluorescence) of hyphae of the *cna1*Δ (SCCMP1M4) and *crz1*Δ (MKY380) mutants and their respective reference strains (SC5314 and CAI-4/CIp10). Filamentation was induced by incubating yeast cells for 3 h at 37 °C in 20% FBS, followed by staining with PI. Arrows indicate PI-positive hyphae. Scale bars: 10 μm. b): Comparison of the percentage of filaments showing PI staining after growth in FBS in mutants lacking the *CNA1*, *CRZ1,* or *PEF1* gene next to their respective control strains. The black bars show mean values with errors (Std Dev) from 3 to 7 technical replicates derived from 2 independent experiments per strain. c): Localization of GFP-Pef1 in hyphae of the *cna1*Δ (MW-Ca134) and *crz1*Δ (MW-Ca132) mutants and their respective control strains SC5314 and CAI-4 expressing the fluorescent fusion protein (MW-Ca124 and MW-Ca125). After incubation for 3 h at 37 °C in 20% FBS, the protein is focused at the hyphal tips of both control strains and the *crz1*Δ mutant (solid arrows), whereas several punctate signals appear in a wider region at the hyphal tip in the *cna1*Δ mutant (dotted arrows). Scale bars: 5 μm. d) Percentage of filaments from the strains presented in panel c showing a focused localization of GFP-Pef1 at the hyphal tip. The black bars show the mean values with errors (Std Dev) from 2 to 3 technical replicates per strain. e) Quantitative analysis of the number of GFP-Pef1 puncta in the hyphal tip region of the strains shown in panel c. The scatter plot represents the mean values and error bars (Std Dev) of the number of fluorescent signals scored in each hyphal tip region (open circles) among the total number (*n*) of filaments for each strain. Statistically significant differences between the mean values shown in b, d, and e (***P < 0.001; ns, not significant) were assessed by one-way ANOVA analysis with Tukey's correction for multiple comparisons.

C. *albicans* mutants lacking a functional Ca^2+^/calcineurin-Crz1 signaling pathway are impaired in cell integrity during normal growth and are hypersensitive to membrane-damaging compounds (Sanglard et al. 2003; Onyewu et al. 2004; Karababa et al. 2006). Consistent with previous studies on the importance of Cna1, the catalytic subunit of the calcineurin phosphatase, for *C. albicans* survival in serum (Sanglard et al. 2003), hyphae of the *cna1*Δ mutant predominantly stained positive with PI when grown in serum, with levels of staining exceeding those observed for *pef1*Δ (Fig. 3a and b). In contrast, the filaments of the *crz1*Δ mutant, which lacks the transcription factor downstream of calcineurin (Karababa et al. 2006), were indistinguishable from those of the control strains, SC5314 and CAI-4/CIp10 (Fig. 3a and b). These observations indicate that the processes mediating membrane integrity during normal filamentous growth of *C. albicans* in serum are dependent on Pef1 and calcineurin, but independent of Crz1-mediated gene expression.

To identify a functional link between calcineurin and Pef1, we expressed GFP-Pef1 during hyphal growth of the *cna1*Δ and *crz1*Δ mutants. In hyphae of the SC5314 and CAI-4 control strains, localization of GFP-Pef1 in the tip region was the same as that previously observed (Figs. 2a and 3c). GFP-Pef1 also localized to the hyphal tip in both the *cna1*Δ and *crz1*Δ mutants but, in the calcineurin-deficient strain, its fluorescence was strongly reduced (Fig. 3c and d). Strikingly, GFP-Pef1 also appeared as dispersed puncta around the hyphal apex of *cna1*Δ (Fig. 3c and e). Thus, the contrasting hyphal integrity phenotypes of the *cna1*Δ and *crz1*Δ mutants correlated with differential localization patterns of GFP-Pef1. These observations suggest that calcineurin activation could be partially responsible for the localization of Pef1 during hyphal growth. Alternatively, loss of calcineurin allows the development of membrane damage that requires differential localization of Pef1 activity for its repair.

### Pef1 puncta are redistributed within the plasma-membrane on exposure to membrane-damaging compounds

**Fig. 4.**
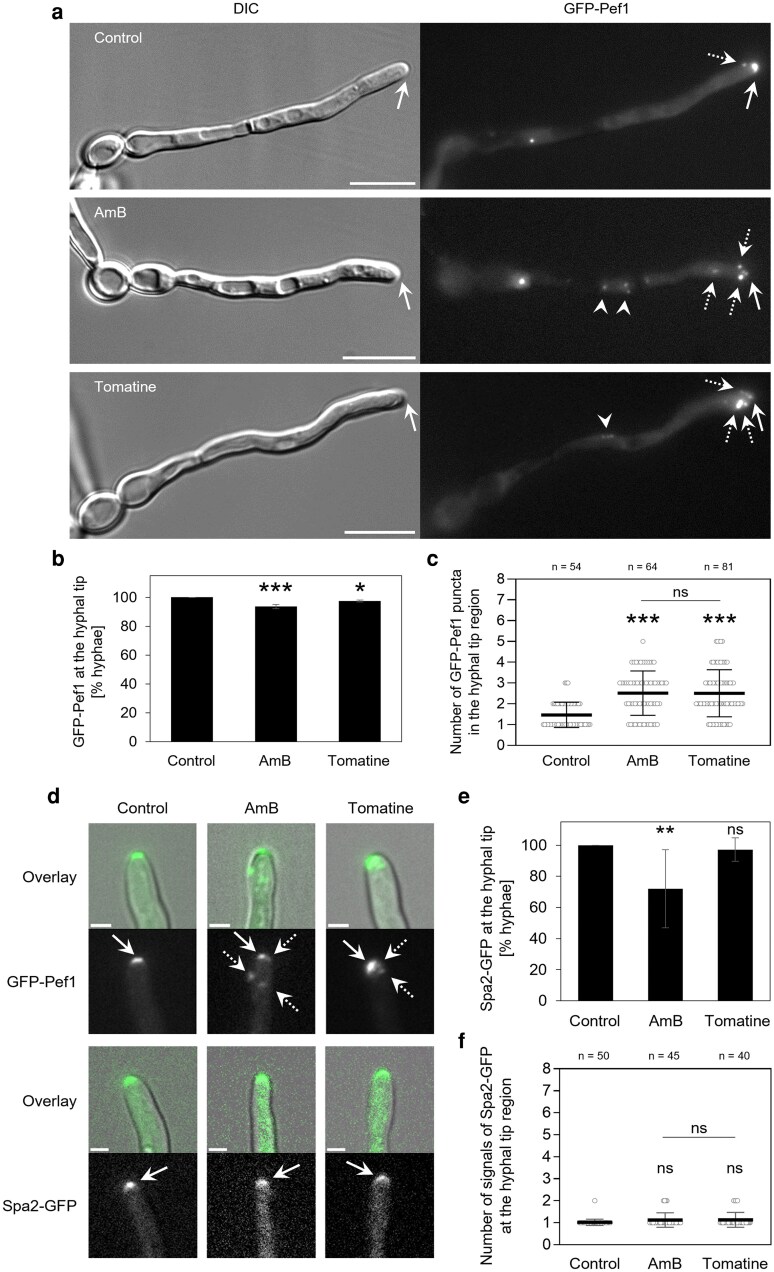
Membrane-disrupting antifungal compounds alter the hyphal tip localization of Pef1 but not the polarisome protein Spa2. a) Localization of GFP-Pef1 (reporter strain MW-Ca58) in hyphae when cells were exposed to 2 μg/mL amphotericin B (AmB) or 50 μg/mL tomatine. Filaments were grown for 3 h at 37 °C in 20% FBS prior to treatment. Images were captured between 5 and 15 min of antifungal exposure. In hyphae of the control strain, GFP-Pef1 appeared at the hyphal tip region (dotted arrows), and in regions distal to the hyphal tip (arrowheads). Scale bars: 10 μm. b): Quantification of the percentage of filaments as shown in panel A, with a focused localization of GFP-Pef1 at the hyphal tip. In the control and during the treatment with AmB or tomatine, almost all filaments show a fluorescent signal of GFP-Pef1 at the polarized cell tips. Bars = mean ± SD, *n* = 2 to 4. c): Quantitative analysis of the number of distinct signals (puncta) of GFP-Pef1 in the hyphal tip region in the absence or presence of AmB or tomatine. From the filaments analyzed in panel B, the number of puncta scored for each hyphal tip (open circles) is represented in a dot plot, including mean values and error bars (Std Dev) from the total number (*n*) of hyphae analyzed per condition. Statistically significant differences in b and c (***P < 0.001; *P < 0.05; ns, not significant) were assessed by one-way ANOVA analysis with Tukey's correction for multiple comparisons. d). Comparison of the localization patterns of Spa2-GFP (WYZ6) with GFP-Pef1 in hyphae treated as above. In contrast to GFP-Pef1, antifungal treatment does not affect Spa2-GFP localization. Overlay: merged DIC and fluorescence images. Scale bars: 2 μm. e): Quantification of the localization of Spa2-GFP at the hyphal tip of filaments, as shown in d. Bars = mean ± SD values from 3 to 12 filaments per 8 to 9 technical replicates per condition. f): Quantitative analysis of the signal distribution of Spa2-GFP at the hyphal tip in the absence and presence of AmB or tomatine. The dot plot shows the number of distinct fluorescent signals observed at the hyphal tip region of each filament (open circles) as well as the mean ± SD from the total number (*n*) of filaments analyzed per condition. Differences were assessed by one-way ANOVA with Tukey's correction for multiple comparisons (**P < 0.01; ns, not significant).

To explore the requirement for Pef1 redistribution to undertake localized membrane repair further, we exposed *C. albicans* hyphae to the membrane-interacting compounds, amphotericin B (AmB, a polyene), or tomatine (a saponin). In both cases, the intensity of the GFP-Pef1 signal at the hyphal tip was reduced and the signal fragmented into puncta within the apical zone (Fig. 4a–c). Moreover, additional puncta of GFP-Pef1 appeared in subapical regions. Overall, these observations suggest that Pef1 localizes to sites of dynamic membrane re-organization during growth but can be redistributed to points throughout the plasma-membrane during chemically induced whole-cell membrane disruption.

As the localization pattern of Pef1 to the hyphal tip during polarized growth is very similar to that seen for polarisome components, we next tested whether the polarisome marker, Spa2, is also redistributed in response to chemical insult (Zheng et al. 2003). Both proteins showed a similar polarized localization in untreated hyphae, but, unlike GFP-Pef1, Spa2-GFP distribution was unaffected by the membrane-targeting compounds, albeit with a slightly reduced signal in AmB-treated hyphae (Supplementary Fig. 4d–f). These observations suggest that, while both Spa2 and Pef1 localize to the hyphal apex during polarized growth, this is achieved via different mechanisms and Pef1 is unlikely to be a constitutive member of the polarisome complex.

### Pef1 function is required for resistance to membrane-damaging compounds

**Fig. 5.**
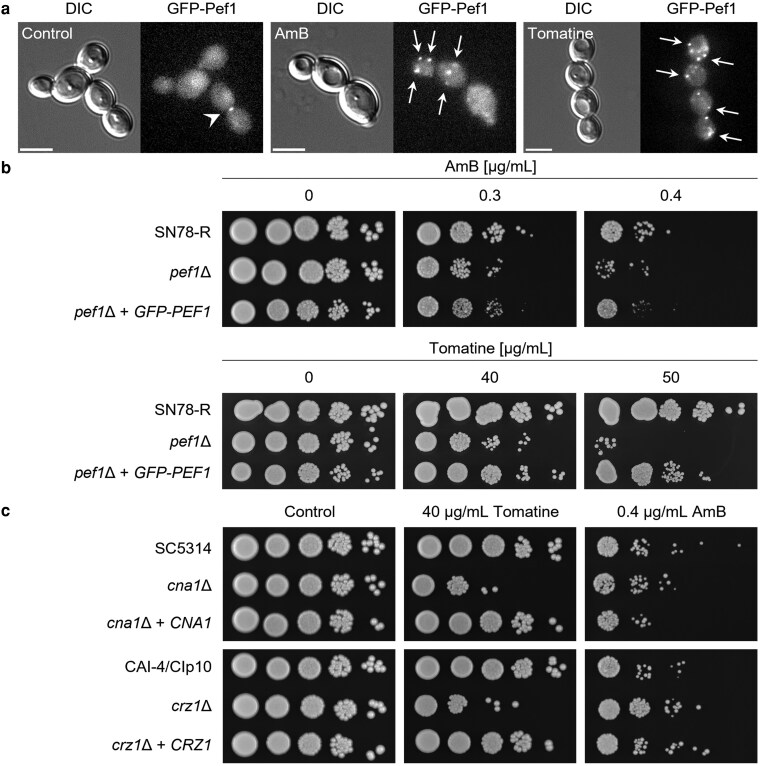
Pef1 and Ca^2+^/calcineurin signaling contribute in a similar manner to the adaptation to membrane-disrupting fungicides. a): Localization of GFP-Pef1 (reporter strain MW-Ca58) during the treatment of yeast cells with antifungal compounds. Yeast cells from YPD cultures were washed in SD medium supplemented with 5 mM Ca^2+^ prior to the exposure to 2 μg/mL AmB, 50 μg/mL tomatine, or 0.125% DMSO (control). Images were captured after 5 to 15 min of antifungal treatment. While GFP-Pef1 localizes at sites of cell polarity in the control (arrowhead; see also Fig. 1d), the protein accumulates at the cell periphery and at intracellular sites during antifungal treatment (arrows). Scale bars: 5 μm. b): Colonies grown from 10-fold serial dilutions of yeast cells of the control strain SN78-R (MW-Ca81), the *pef1*Δ mutant (MW-Ca27), and the complemented strain (MW-Ca58) spotted on solid YPD medium with and without the indicated concentrations of AmB or tomatine. Growth was captured after 2 d of incubation at 37 °C. c): Ten-fold serial dilutions of yeast cells from the *cna1*Δ (SCCMP1M4) and *crz1*Δ (MKY380) mutants, as well as the respective control strains (SC5314, CAI-4/CIp10) and complemented mutants (SCCMP1MK2, MKY381), were spotted onto YPD agar plates with and without tomatine or AmB and incubated for 2 d at 37 °C.

We next examined the distribution of GFP-Pef1 in yeast cells during treatment with AmB or tomatine. Similar to our observations in hyphae, exposure to the polyene and saponin induced the formation of fluorescent puncta throughout the peripheral membrane of yeast cells (Fig. 5a). Thus, both types of ergosterol-binding compounds dramatically altered the localization pattern of Pef1 in yeast and hyphae, consistent with its recruitment to sites of chemically-induced membrane disruption.

We also evaluated the importance of Pef1 function in countering their antifungal effect and compared this with the requirement for calcineurin and the Crz1 transcription factor. Spot assays showed that the *pef1*Δ mutant was only mildly susceptible to AmB compared to the control strains (Fig. 5b). In a similar manner, there was no change in susceptibility to the polyene-derivative, nystatin, even though this commonly-used topical antifungal also induced the formation of multiple puncta of GFP-Pef1 in yeast cells (Supplementary Fig. 6a and b). In contrast, *pef1*Δ showed clear susceptibility to tomatine (Fig. 5b). Interestingly, the mutant shared these different fungicide susceptibility phenotypes with both mutants of the calcineurin signaling pathway (Fig. 5c). Taken together, these results demonstrate a differential role of Pef1 in *C. albicans* in response to membrane-disrupting compounds that is comparable to that of PEF1 in *N. crassa* (Schumann et al. 2019), indicating that orthologous PEF-hand proteins in distantly related fungal species mediate a conserved response to membrane damage.

### Pef1 is required for virulence in the *Galleria mellonella* infection model

**Fig. 6.**
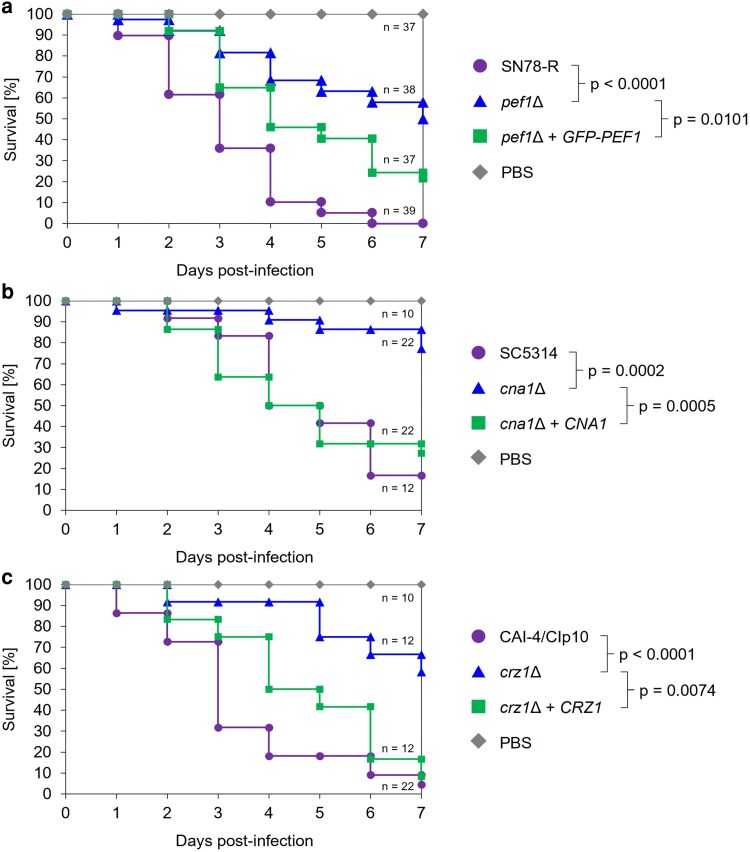
Loss of Pef1 attenuates the virulence of *C. albicans.* Survival plots of *G. mellonella* larvae infected with yeast cells of SN78-R (MW-Ca81), compared to a) the *pef1*Δ mutant (MW-Ca27), b) the *cna1*Δ mutant, or c) the *crz1*Δ mutant. After injection with 1 × 10^6^ yeast cells (prepared in phosphate-buffered saline, PBS) or with sterile PBS as a control, the larvae were incubated for 7 d at 37 °C and checked daily for survival. The plots represent pooled data from 5 independent experiments with the indicated total number (*n*) of larvae used per strain. Comparisons of survival plots were statistically assessed by Log-rank analysis, resulting in the indicated *P* values.

To determine whether Pef1 function is required for fungal pathogenicity, we tested the virulence of the *pef1*Δ mutant in the larvae of the greater wax moth, *Galleria mellonella*, a validated model for fungal virulence studies (Curtis et al. 2022). This model features innate immune responses that include the release of membrane-targeting antimicrobial peptides (Sheehan and Kavanagh 2019; Tsai et al. 2016). One week after infecting groups of larvae with *C. albicans*, the population death rate for the *pef1*Δ mutant was only ∼50%, significantly lower than the 100% and ∼ 80% of the larvae infected with SN78-R and the complemented *pef1*Δ+GFP-PEF1 strains, respectively (*P* ≥ 0.0001) (Fig. 6a). By comparison, observed death rates for the *cna1*Δ and *crz1*Δ mutants, were ∼20% and ∼40%, respectively, compared to ∼ 50 to 90% in the corresponding control strains (Supplementary Fig. 6b and c). This is consistent with the impaired virulence reported for these mutants in murine models of systemic candidiasis (Sanglard et al. 2003; Karababa et al. 2006; Bader et al. 2003, 2006). Loss of Pef1 therefore attenuates the pathogenicity of *C. albicans* in this infection model to similar levels seen for mutants defective in the calcineurin signaling pathway. This supports the idea that Pef1 plays an important role in maintaining membrane integrity, both at polarity sites and during membrane stress imposed by external factors.

## Discussion

Fungal PEF-hand proteins have previously been associated with polarized growth during budding in *S. cerevisiae* yeast and cell membrane repair in germlings and filaments of *N. crassa* and *B. cinerea* (Schumann et al. 2019; You et al. 2024; Vernarecci et al. 2007). Our study suggests that the orthologous Pef1 protein in *C. albicans* may share both functions through supporting membrane integrity at sites of dynamic membrane re-organization, such as during polarized growth and septum formation in both yeast and hyphae of this polymorphic species. Our study contributes to the growing evidence for a highly conserved role of eukaryotic PEF-hand proteins in response to membrane perturbation, which in fungi is currently best understood in the context of membrane-targeting antifungal compounds that trigger the fast and dynamic recruitment of these Ca^2+^-binding proteins to the cell membrane. In addition, Pef1 in *C. albicans* takes an additional role at sites of polarized growth, which seems to be specific for fungal species with a yeast morphotype. Pef1 appears to act as a specialist “first responder” that stabilizes the plasma membrane at sites of perturbation, where immediate repair is required to maintain cell viability. Its function in *C. albicans* is therefore relevant not only to normal growth, but also to survival of immune-system attack and resistance to membrane-targeting antifungal drugs.

### The *C. albicans* PEF-hand protein Pef1 is required for membrane integrity during hyphal growth

In contrast to filamentous fungi, in which PEF-hand proteins reside in the cytosol during normal hyphal growth (Schumann et al. 2019; You et al. 2024), the *C. albicans* Pef1 protein was stably localized at the polarized hyphal tip when growing in the filamentous morphotype. In *S. cerevisiae* yeast cells, Pef1 activity was proposed to be daughter-cell specific and the protein was recruited to sites of cell polarity and septa during budding (Vernarecci et al. 2007). Pef1 function appears to be similar in *C. albicans* yeast, but here we gained further insights by observing its role in hyphae. Relevant to its role as a pathogen, a striking finding was that the viability of hyphae of the *C. albicans pef1*Δ mutant was compromised during polarized growth in 20% serum, but not in agar plates containing 10% serum or in other lab-derived media. As *pef1*Δ hyphae partially mimicked the loss-of-integrity phenotype of the *cna1*Δ mutant, in which inhibition of Ca^2+^ influx channels is relieved, this suggests that these proteins act in the same stress–response pathway. Consistent with previous research, calcineurin is required for the survival of *C. albicans* and other *Candida* species in serum, which represents a form of Ca^2+^ ion stress (Sanglard et al. 2003; Blankenship et al. 2003, Blankenship and Heitman, 2005; Reedy et al. 2010; Chen et al. 2011). Interestingly, we found that only the *cna1*Δ, but not the *crz1*Δ, mutant was deficient in maintaining membrane integrity during hyphal growth in serum, and that these different phenotypes correlated with different localization patterns of Pef1 at the hyphal tip. These observations suggest important roles of Crz1-independent calcineurin activity and Pef1 function during normal hyphal growth in serum. Although the *cna1*Δ mutant shared this defect with the *pef1*Δ mutant, the much larger degree of impaired hyphal integrity by loss of Cna1 function may reflect a broader role for this protein in not only Ca^2+^/calcineurin signaling but in limiting Ca^2+^ entry into the cell (Park et al. 2019). Since the *pef1*Δ mutant was barely impaired by cell wall stress, we conclude that Pef1 may be just one of many proteins whose function is linked to, if not directly regulated by, calcineurin activity. This is consistent with the idea that Pef1 is redistributed within the membrane when calcium influx inhibition is lost in the *cna1*Δ mutant (Fig. 3c–e). The absence of a GFP-Pef1 signal in sub-apical compartments in the *cna1*Δ mutant or on exposure to membrane-stressors, which impacts all cells, is consistent with the observation that Pef1 expression is confined to daughter cells (Vernarecci et al. 2007).

### Fungal PEF-hand proteins share a conserved function in maintaining cell membrane integrity during membrane disruption by antifungal compounds

All so far studied PEF-hand proteins in fungi share a conserved role in the response and adaptation to external conditions that cause acute membrane disruption. Our results in *C. albicans* support previous findings in *N. crassa* and *B. cinerea* that showed speedy membrane recruitment of these Pef1 orthologs after treatment with a polyene or saponin (Schumann et al. 2019; You et al. 2024). Although Pef1 is constitutively associated with polarized membrane sites in yeast and hyphal cells of *C. albicans*, the dramatic change in its subcellular localization upon exposure to membrane-disrupting fungicides is consistent with a role in mediating a membrane stress response. The localization patterns of GFP-Pef1 in dividing yeast cells and at the growing hyphal tip might therefore reflect intrinsic membrane stress during normal growth. We propose that the altered localization of Pef1 during antifungal-induced stress represents a sudden shift from guarding membrane integrity during polarized growth towards initiating a repair response after membrane attack.

The dynamic recruitment of fungal PEF-hand proteins reflects the intensity and severity of membrane attack by fungicides that directly interact with ergosterol, which is highly enriched in fungal tips (Puumala et al. 2024; Augustin et al. 2011; You et al. 2024; Serrano et al. 2023; Alvarez et al. 2007; Weichert et al. 2020; Takeshita et al. 2008). However, mutants that lack PEF-hand proteins in different species share the same differential phenotypes in the presence of polyenes and saponins. Similar to the filamentous fungi, the *pef1*Δ mutant of *C. albicans* did not show increased susceptibility to nystatin or AmB despite the membrane recruitment of Pef1 in response to these polyenes. In contrast, Pef1 was required for the tolerance of tomatine, a saponin compound that this human microbiome-associated fungus unlikely encounters in the host environment. The mismatch between the subapical relocalization of Pef1 and the ability of the cell to withstand membrane-targeting drugs may reflect the extent or specific types of damage caused by these reagents, where Pef1 is recruited to sites of damage but its activity is not sufficiently effective to mount a suitable response. Similar observations in distantly related fungal species promote the idea that the molecular basis of Pef1-mediated membrane stress adaptation in fungi is highly conserved despite their different ecologies and lifestyles. It is possible that *C. albicans* encounters other metabolites or molecules present in its niches, either originating from host cells or microbes, that have membrane-disrupting modes of action similar to those of saponins, which could explain the conservation of this response in this species.

Many membrane-targeting compounds, whether causing the formation of small or large membrane pores or ruptures, invoke a sudden influx of external Ca^2+^ into the cytosol. An example is the speedy recruitment of the human ALG-2 protein to membrane wounds that cause an increase in cytosolic Ca^2+^ (Scheffer et al. 2014; La Cour et al. 2018). In *N. crassa*, the availability of external Ca^2+^ is also required for the membrane recruitment of PEF1 (Schumann et al. 2019), implying that this basic function of PEF-hand proteins is likely conserved in all eukaryotic cells. Given the strong conservation of the Ca^2+^-binding helix-loop-helix motifs in human and fungal PEF-hand proteins, all fungal orthologs are likely to act in a similar manner as Ca^2+^-sensor proteins, as previously established for ALG-2. Interestingly, it was recently shown that Ca^2+^ directly stimulates the membrane binding of ALG-2 by neutralizing electrostatic repulsion with acidic phospholipid membranes (Shukla et al. 2024). This Ca^2+^ binding mechanism might also mediate the membrane association of the fungal PEF-hand proteins, given the strong conservation of the EF-hand motifs. Moreover, the disordered N-termini of fungal ALG-2 orthologs might also allow for a direct or indirect membrane association, since these regions are known to enable close membrane associations and the interaction with other proteins directly tethered to the lipid bilayer (Cornish et al. 2020). ALG-2 does not feature such a disordered N-terminal extension, but its interaction partner, peflin, does (Sargeant et al. 2021; Kitaura et al. 2001). While it is unknown whether peflin is directly associated with membrane repair, we hypothesize that important differences in the mobilization, interactions, and functions of fungal and human PEF-hand proteins exist.

### Pef1-mediated membrane integrity is a specific subset of Ca^2+^-dependent mechanisms that support cellular homeostasis

In addition to the membrane recruitment of PEF-hand proteins, a rapid rise in cytosolic Ca^2+^ levels also activates Ca^2+^/calcineurin signaling, which mediates a broad adaptive membrane stress response in fungi (Park et al. 2019). Since Ca^2+^ influx is a common signal, we reasoned that Pef1 function is, at least in part, related to this major stress response pathway. Our results showed that the *pef1*Δ mutant shares fungicide-related phenotypes with mutants defective in Ca^2+^/calcineurin signaling. Loss of either Cna1 or Crz1 caused hypersensitivity to tomatine, but not to AmB, which largely resembled the phenotypes of the *pef1*Δ mutant. This implies that canonical Ca^2+^/calcineurin signaling and Pef1 function are individually dispensable for survival during the exposure to polyenes, but each process is required for the tolerance of saponin-induced membrane disruption. We assume that other forms of direct membrane disruption also induce both mechanisms, which jointly operate to reach the full scale in the response to membrane attack.

Exploring cellular Ca^2+^ dynamics during membrane stress with real-time reporter systems, such as the genetically-encoded Ca^2+^ indicator GCaMP6 (Giuraniuc et al. 2023; Muñoz et al. 2020), would improve our mechanistic understanding on how Ca^2+^-dependent processes specifically mediate the adaption to growth conditions and antifungal compounds that challenge membrane integrity. Moreover, the expression of GFP-Pef1 in additional sets of mutants and clinical isolates of *C. albicans* that show different membrane stress phenotypes would complement studies on Ca^2+^ dynamics and collectively expand our knowledge on Ca^2+^-dependent mechanisms of membrane integrity.

### Pef1 contributes to *C. albicans* virulence

The roles identified here for Pef1 in polarized growth and membrane integrity appear to play a role in the pathogenicity of *C. albicans*. The filamentous growth of this human pathogen is a major determinant of its pathogenic potential (Kumamoto et al. 2020; Austermeier et al. 2020; Westman et al. 2019) and the host environment contains factors that can directly disrupt microbial membranes, in particular membrane-targeting antimicrobial peptides (Struyfs et al. 2021; Curtis et al. 2022). Our observation that loss of Pef1 significantly attenuated the killing of infected insect larvae identifies this PEF-hand protein as a factor contributing to the pathogenic potential of *C. albicans*. However, compared to mutants defective in Ca^2+^/calcineurin signaling, this pathogenicity defect of the *pef1*Δ mutant appears less pronounced, consistent with the broader role of calcineurin as a regulator of signaling in cellular stress adaptation (Park et al. 2019) whereas Pef1 plays a specific effector role in response to direct membrane stress. Nonetheless, Pef1 and its repair mechanisms contribute significantly to pathogenicity under specific circumstances. In turn, human cells employ an ALG-2-initiated membrane repair process when attacked by candidalysin (Westman et al. 2022). This indicates that during fungal infection, a protective response towards the reciprocal usage of membrane attack involves defense mechanisms mediated by PEF-hand proteins with conserved functions.

Strong evidence supporting a model for the concerted activation of preformed and induced molecular factors that protect the cell membrane has recently been presented in *B. cinerea* during its interaction with tomato plants (You et al. 2024). While the membrane-targeting host defense compound tomatine triggers as a first-level response the quick recruitment of the BcPEF1 ortholog to the fungal membrane, the induction of several membrane-modifying and tomatine-detoxifying enzymes required transcriptional activation. However, the availability of these second-level mechanisms to protect the fungal membrane strongly promoted tomatine tolerance and plant pathogenicity in a mutant strain lacking BcPEF1 (You et al. 2024). Thus, progressively unfolding different mechanisms of membrane integrity can boost the pathogenic potential of fungi. It remains to be investigated which molecular mechanisms other than the Pef1-mediated response are involved in the adaptation of *C. albicans* to membrane stress and adaptation to antifungal drugs. Considering the increasing incidence of resistance to antifungal drugs (Perlin et al. 2017), the molecular dissection of membrane integrity mechanisms could inform novel strategies to combat hard-to-treat fungal infections. Further research on membrane repair in fungi could identify currently unknown points of vulnerability on the membrane level of pathogenic species, which might be exploited to design novel specific or combinatorial treatment options against fungal infections, and to prevent and combat antifungal resistance.

## Materials and methods

### Standard growth conditions, media and reagents

Yeast cell suspensions of *C. albicans* were grown as standard overnight cultures at 30 °C and 200 rpm in YPD broth (1% [w/v] yeast extract [BD Difco], 2% [w/v] bacto-peptone [BD Difco], 2% [w/v] glucose), unless otherwise stated. Cell concentrations were photometrically determined as optical density at 600 nm (OD_600_). Prototrophic and auxotrophic strains were selected on synthetic defined (SD) medium (0.67% [w/v] yeast nitrogen base [YNB; BD Difco], 2% [w/v] glucose, 1.5% [w/v] agar) with or without the supplementation of histidine, leucine, and/or uridine (each at 200 µg/mL), respectively. To induce hypha formation, yeast cells were washed in sterile MilliQ (MQ) water or double-distilled water and incubated at 37 °C in 20% (v/v) fetal bovine serum (FBS; Sigma) using 8-well polymer chamber slides with ibiTreat surface modification (ibidi). Stock solutions of antifungal compounds and stress agents were prepared as follows: 10% (w/v) SDS in MQ water; 10 mg/mL CFW (fluorescence brightener 28, Sigma) in MQ water; 1 to 2 mg/mL AmB (Sigma) in DMSO; 50 mg/mL nystatin (Millipore) in DMSO; 20 or 25 mg/mL tomatine (biorbyt or Carl Roth) in either DMSO (for microscopy) or methanol with 0.2% (v/v) formic acid (for plate assays). For molecular cloning of plasmids, electro-competent or chemo-competent *Escherichia coli* strains were grown at 37 °C in Luria-Bertani medium (1% [w/v] tryptone [BD Difco], 0.5% [w/v] yeast extract [BD Difco], 0.5% [w/v] NaCl) supplemented with 100 µg/mL ampicillin or 50 µg/mL kanamycin. Standard protocols were used for plasmid DNA manipulation, bacterial transformations, and the preparation of plasmid DNA from bacterial cell suspensions or genomic DNA from fungal yeast cell suspensions.

### *C. albicans* strain construction

All *C. albicans* strains used and constructed in this study are listed in Table 1. Oligonucleotides (primers) used for molecular cloning and confirmatory PCR analyses are listed in Supplementary Table 1.

**Table 1.** Strains of *C. albicans* used in this study.

Name	Identifier	Genotype	Origin
SC5314	–	Prototrophic wild-type strain (clinical isolate)	Gillum et al., 1984, *Mol Gen Genet*
CAI-4	–	*ura3*Δ-*iro1*Δ::*imm^434^*/*ura3*Δ-*iro1*Δ::*imm^434^*	Fonzi and Irwin, 1993, *Genetics*
SN78	–	*his1*Δ/*his1*Δ, *leu2*Δ/*leu2*Δ, *ura3*Δ-*iro1*Δ::*imm^434^*/*ura3*Δ-*iro1*Δ::*imm^434^*	Noble and Johnson 2005, *Euk Cell*
*pef1*Δ Ura^−^ (isolate #1)	MS-01	As SN78 but *pef1*Δ::*CdHIS1*/*pef1*Δ::*CmLEU2*	This study.
*pef1*Δ Ura^−^ (isolate #2)	MS-02	As SN78 but *pef1*Δ::*CdHIS1*/*pef1*Δ::*CmLEU2*	This study.
*pef1*Δ Ura^−^ (isolate #3)	MS-03	As SN78 but *pef1*Δ::*CdHIS1*/*pef1*Δ::*CmLEU2*	This study.
SN78-R	MW-Ca81	As SN78 but *rps1*Δ::(*CaHIS1*, *CaURA3*, *CaLEU2*)/*RPS1*	This study.
*pef1*Δ	MW-Ca27	As MS-1 but *rps1*Δ::*URA3*/*RPS1*	This study.
*pef1*Δ + *GFP-PEF1*	MW-Ca58	As MS-1 but *rps1*Δ::(*pACT1-GFP-PEF1*, *URA3*)/*RPS1*	This study.
*cna1*Δ	SCCMP1M4	As SC5314 but *cna1*Δ::*FRT*/*cna1*Δ::*FRT*	Bader et al. 2006, *Infection and Immunity*
*cna1*Δ + *CNA1*	SCCMP1MK2	As *cna1*Δ/Δ but *cna1*Δ::*FRT*/*cna1*Δ::*FRT*+*CNA1*	Bader et al. 2006, *Infection and Immunity*
CAI-4/CIp10	–	As CAI-4 but *rps1*Δ::CIp10/*RPS1*	Brand et al. 2004, *Euk Cell*
*crz1*Δ	MKY380	*crz1*Δ::*hisG*/*crz1*Δ::*hisG*, *ura3*Δ-*iro1*Δ::*imm^434^*/*ura3*Δ-*iro1*Δ::*imm^434^*, *rps1*Δ::*URA3*/*RPS1*	Karababa et al. 2006, *Molecular Microbiology*
*crz1*Δ + *CRZ1*	MKY381	As *crz1*Δ/Δ but *crz1*Δ::*CRZ1*/*SAT1*	Karababa et al. 2006, *Molecular Microbiology*
*crz1*Δ Ura^−^	MKY59	*crz1*Δ::*hisG*/*crz1*Δ::*hisG*, *ura3*Δ-*iro1*Δ::*imm^434^*/*ura3*Δ-*iro1*Δ::*imm^434^*	Karababa et al. 2006, *Molecular Microbiology*
SC5314 + *GFP-PEF1*	MW-Ca124	As SC5314 but *rps1*Δ::(*pACT1-GFP-PEF1*, *NAT1*)/*RPS1*	This study.
*cna1*Δ + *GFP-PEF1*	MW-Ca134	As *cna1*Δ/Δ but *rps1*Δ::(*pACT1-GFP-PEF1*, *NAT1*)/*RPS1*	This study.
CAI-4 + *GFP-PEF1*	MW-Ca125	As CAI-4 but *rps1*Δ::(*pACT1-GFP-PEF1*, *URA3*)/*RPS1*	This study.
*crz1*Δ + *GFP-PEF1*	MW-Ca132	As *crz1*Δ/Δ Ura^−^ but *rps1*Δ::(*pACT1-GFP-PEF1*, *URA3*)/*RPS1*	This study.
Spa2-GFP	WYZ6	*spa2*Δ::*ARG4*/*spa2*::*SPA2-GFP, rps1*Δ::*URA3*/*RPS1*	Zheng et al. 2003, *Molecular Microbiology*

#### Deletion of the PEF1 gene

To delete both alleles of the *PEF1* gene in the triple-auxotrophic (His*^−^*, Leu*^−^*, Ura*^−^*) reference strain, SN78, we employed a previously published gene deletion strategy (Noble and Johnson 2005). Using genomic DNA from strain SC5314 as a template, left (5′) and right (3′) flanks homologous to ∼350-bp sequences upstream and downstream of the *PEF1* gene were PCR-amplified with the primer pairs 1,052/1,053 and 1,054/1,055, respectively. The selectable markers *LEU2* (from *Candida maltose*, *CmLEU2*) and *HIS1* (from *Candida dubliniensis*, *CdHIS1*) were PCR-amplified from plasmids pSN40 and pSN52, respectively, with the primer pair 1,050/1,051 as described before (Noble and Johnson 2005). To generate both *PEF1* knock-out (KO) cassettes, the 5′ and 3′ flanks were combined with the *LEU2* or *HIS1* marker using a fusion PCR approach (Noble and Johnson 2005). Yeast cells of strain SN78 were prepared for transformation with the KO cassettes using a lithium acetate/DTT protocol and transformed by electroporation as described before (Thompson et al. 1998, De Backer et al. 1999). First, the *LEU2*-containing KO cassette was transformed into SN78 followed by selection of heterozygous mutants on SD agar plates supplemented with uridine and lacking leucine. Second, one of these heterozygous isolates was further transformed with the *HIS1*-containing KO cassette, resulting in homozygous *pef1*Δ Ura^−^ mutants (MS-01 to MS-03) that were selected on uridine-containing SD agar plates lacking both leucine and histidine (Supplementary Fig. 2a and b). Strain MS-01 was transformed with the StuI-linearized plasmid CIp10 to integrate a copy of *URA3* at the *RPS1* locus as describe before (Murad et al. 2000; Brand et al. 2004), resulting in the prototrophic *pef1*Δ mutant strain, MW-Ca27 (Supplementary Fig. 2c). To restore prototrophy in the triple-auxotrophic parental strain SN78, plasmid CIp20, which contains the markers *URA3* and *HIS1* from *C. albicans* (Dennison et al. 2005), was linearized with SacI and NotI to allow for the integration of the *LEU2* marker from *C. albicans* (*CaLEU2*, including ∼850 bp of its promoter and ∼200 bp of its terminator regions in analogy to *CmLEU2* from pSN40). The *CaLEU2* marker was PCR-amplified from SC5314 with the primer pair 2024/2025. In line with the CIp series of vectors for integration at the *C. albicans RPS1* locus (Murad et al. 2000; Dennison et al. 2005), the resulting plasmid was named CIp40 (lab identifier 1122/B342; Supplementary Fig. 2d). Strain SN78 was transformed with StuI-linearized CIp40, as described above, to generate the prototroph SN78-R (strain MW-Ca81). After selection on SD agar plates lacking leucine, histidine and uracil/uridine, integration of CIp40 at the *RPS1* locus was confirmed by PCR (Supplementary Fig. 2d).

#### GFP-tagging of Pef1

The *pef1*Δ Ura^−^ mutant (strain MS-01) was complemented with a construct that encodes Pef1 fused at its N-terminus to the *C. albicans*-codon optimized sequence of the green fluorescent protein (GFP) for live-cell imaging and fluorescence microscopy (Cormack et al. 1997). Plasmid pExpArg-pACT1GFPRID (Corvest et al. 2013) was stepwise modified for expression of the *GFP-PEF1* construct under the *ACT1* promoter (*ACT1*p) (Barelle et al. 2004). First, the XbaI and SalI sites were used to replace the *ARG4* marker by the *URA3* gene from SC5314, which was PCR-amplified with the primer pair 1,953/1,954 (resulting in the intermediate plasmid pExpUra-pACT1GFPRID, lab identifier 1102). Next, the *RID* reporter was excised via RsrII and MluI and replaced by the *PEF1* gene including 500 bp of the sequence downstream of its stop codon, which was PCR-amplified from SC5314 with the primer pair 1,955/1,956. The resulting plasmid pExpUra-pACT1-GFP-PEF1 (lab identifier 1104/B341; Supplementary Fig. 2e) and linearized with StuI prior to transformation as described above. Transformants were selected on SD agar plates without uracil/uridine. *GFP-PEF1* integration at the RPS1 locus was confirmed by PCR (Supplementary Fig. 2e) and fluorescence microscopy. Plasmid pExpUra-pACT1-GFP-PEF1 was integrated into the reference strain, CAI-4, and the *crz1*Δ Ura^−^ mutant, and confirmed by PCR. Integration of *GFP-PEF1* into SC5314 and the *cna1*Δ mutant (SCCMP1M4): the *URA3* marker in plasmid pExpUra-pACT1-GFP-PEF1 was excised by NotI and SpeI and replaced by a codon-optimized *NAT1* selectable marker (Shen et al. 2005). This *CaNAT1* marker was PCR-amplified using primer pair MW-P1/MW-P2 from plasmid CIp-NAT, a variant of plasmid CIp10 containing the *TEF1*p*-CaNAT1-TEF1*t construct (Shahana et al. 2014). The resulting plasmid, pACT1-GFP-PEF1-NAT1 (lab identifier 1130/B343), was linearized with StuI and transformed into SC5314 and *cna1*Δ using a 15-min heat shock step at 44 °C followed by a recovery phase in YPD broth supplemented with uridine for 4 h at 30 °C and 200 rpm. Transformants were selected on Sabouraud dextrose agar plates (Oxoid) supplemented with 200 to 400 µg/mL nourseothricin (Jena Bioscience). The *GFP-PEF1*-expressing SC5314 and *cna1*Δ strains were confirmed by PCR and fluorescence microscopy.

### Quantification of yeast cell growth in broth

#### Microtiter plates

Cells were inoculated in 5 mL YPD broth and incubated at 30 °C and 200 rpm overnight. Cells from 1 mL of each preculture were pelleted for 60 s at 3,500 rpm and washed twice in 1 mL of fresh YPD broth. Cell suspensions were diluted to OD_600_ = 0.1, and 200 µL or sterile YPD broth was added to the wells of 96-well flat-bottomed plates (Sarstedt), with 3 to 6 technical replicates per sample. The plate was sealed with a Breathe-Easy sealing membrane (Diversified Biotech) and incubated for 24 h at 30 °C in an Infinite 200 PRO plate reader (TECAN) with constant orbital shaking (amplitude: 3 mm). The OD_600_ was measured at 4 positions per well at 30-min intervals (orbital shaking; duration: 8 s, amplitude: 3 mm). Average growth curves were obtained by normalizing the OD_600_ values to the starting inoculum.

#### Shaking flask cultures

Five milliliters of cells from overnight cultures were washed in fresh YPD and adjusted to OD_600_ = 10. Flasks (250-mL) containing 50 mL fresh YPD broth were inoculated to OD_600_ = 0.1. Flasks were incubated at 37 °C and 200 rpm, and growth was determined by measuring OD_600_ at various timepoints.

### DIC and fluorescence microscopy

Live-cell imaging was acquired on an inverted Zeiss AxioObserver Z1 microscope equipped with DIC and fluorescence setups using a Plan-Neofluor 40X/1.3 numerical aperture oil immersion objective (Carl Zeiss), with a 16-bit CoolSNAP H2 charge-coupled-device (CCD) camera (Photometrics) within an incubation chamber (PeCon GmbH) at 37 °C. Images were acquired with the ZEISS ZEN software and processed in the Fiji freeware (https://fiji.sc/).

#### Analysis of yeast cell budding

Cells from standard overnight cultures were diluted 1:10 in fresh YPD and further incubated at 30 °C and 200 rpm. To test for a potential cell-separation defect, cells were sonicated for 20 cycles at amplitude = 20 for 10 s, followed by 40 s off. Samples were prepared on standard microscope slides and observed by DIC microscopy.

#### Localization of GFP-Pef1

For localization studies in the yeast morphotype, cells were first diluted 1:100 in fresh YPD broth, further incubated at 30 °C and 200 rpm for about 5 h, and diluted 1:10 in SD medium supplemented with 5 mM Ca^2+^. Prior to preparing samples for fluorescence microscopy, aliquots of the cells were treated with 2 µg/mL AmB, 20 µg/mL nystatin, 50 µg/mL tomatine, or 0.2 to 0.5% (v/v) DMSO. Images of the cells were captured within 5 to 15 min after treatment. For hyphal localization of GFP-Pef1, yeast cells were washed in ddH_2_O, resuspended in 20% (v/v) FBS to OD_600_ = 0.01, and seeded into ibidi chambers at 300 µL/well. Hyphae were treated with AmB or tomatine at the above-mentioned final concentrations by adding 100 µL of the compounds or DMSO (4× concentrated in 20% [v/v] FBS) to the wells. Images were captured by fluorescence microscopy within 15 min of antifungal exposure.

#### Staining with PI or FM4-64

Yeast cells were transferred to 20% (v/v) FBS or Spider medium (79) and incubated for 3 to 4 h at 37 °C in ibidi chambers in 300 µL/well. For the staining with PI (stock solution of 1 mg/mL in MQ water), 100 µL of fresh FBS medium or Spider medium containing the dye (4× concentrated in 20% [v/v] FBS) was added to a final concentration of 2 µg/mL. Cells were stained with FM4-64 (stock solution of 100 µg/mL in MQ water) by adding 100 µL of FBS medium supplemented with the dye (4× concentrated in 20% [v/v] FBS) to a final concentration of 0.1 µg/mL. Images were captured by fluorescence microscopy at 5 to 15 min after the addition of each dye.

### Antifungal compound susceptibility testing

The OD_600_ values were measured for overnight cultures in YPD and cell concentrations were adjusted to OD_600_ = 1 in sterile ddH_2_O water. Drops of 5 µL from 10-fold serial dilutions in ddH_2_O were spotted onto solid media with and without AmB (0.1 to 1.0 µg/mL), nystatin (2 to 5 µg/mL), tomatine (20 to 80 µg/mL), SDS (0.01 to 0.04%), CFW (20 to 80 µg/mL), or EGTA (5 to 20 mM). Plates were incubated at 37 °C for 1 to 2 d and images captured.

### Agar invasion assays

Agar invasion was assessed as previously described (Bedekovic et al. 2020). Yeast cells from overnight cultures were diluted to 2.5 × 10^4^ cell per 5-µL spot, and inoculated on Spider agar or 10% (v/v) serum (FBS) agar (1.5% [w/v]) plates. Colony diameters were measured after incubation at 30 °C or 37 °C for 5 to 7 d. To determine the depth of agar invasion, cross-sections of colonies were prepared with a razor blade and imaged against a ruler. For each strain and growth condition, the ratio between the average colony diameters and average penetration depths was calculated.

### Insect larvae infection model

*C. albicans* strains were grown in 5 mL YPD broth for ∼ 22 h at 30 °C and 200 rpm. Cells were diluted 1:100 in fresh YPD and incubated as before. Cells were harvested at 3,000 rpm for 1 min and washed 3 times in PBS buffer prior to determining the cell numbers with a photometer and a hemocytometer. Groups of 10 or 12 similarly sized larvae of *G. mellonella* were infected via the left last proleg with 20 µL of PBS buffer containing 2 × 10^6^ yeast cells or 20 µL of sterile PBS with sterile single-use insulin syringes (30G × 1/2″; B. Braun, Germany). Larvae were kept for 7 d at 37 °C in the dark and survival was monitored daily. Larvae displaying extensive dark-brown pigmentation and loss of motility were scored as dead.

### Statistical data analysis

Statistical analysis was performed using GraphPad Prism version 9 for Windows (GraphPad Software, San Diego, CA, USA).

## Supplementary Material

jkag075_Supplementary_Data

## Data Availability

All data supporting the findings of this study are provided within the manuscript and its Supplementary Materials. All strains generated in this study are available from the corresponding author upon request. Supplemental material available at G3 online.

## References

[jkag075-B1] Alvarez FJ, Douglas LM, Konopka JB. 2007. Sterol-rich plasma membrane domains in fungi. Eukaryot Cell. 6:755–763. 10.1128/EC.00008-07. 17369440 PMC1899238

[jkag075-B2] Athanasopoulos A, André B, Sophianopoulou V, Gournas C. 2019. Fungal plasma membrane domains. FEMS Microbiol Rev. 43:642–673. 10.1093/femsre/fuz022. 31504467

[jkag075-B3] Augustin JM, Kuzina V, Andersen SB, Bak S. 2011. Molecular activities, biosynthesis and evolution of triterpenoid saponins. Phytochemistry. 72:435–457. 10.1016/j.phytochem.2011.01.015. 21333312

[jkag075-B4] Austermeier S, Kasper L, Westman J, Gresnigt MS. 2020. I want to break free—macrophage strategies to recognize and kill *Candida albicans*, and fungal counter-strategies to escape. Curr Opin Microbiol. 58:15–23. 10.1016/j.mib.2020.05.007. 32599492

[jkag075-B5] Bader T, Bodendorfer B, Schröppel K, Morschhäuser J. 2003. Calcineurin is essential for virulence in *Candida albicans*. Infect Immun. 71:5344–5354. 10.1128/IAI.71.9.5344-5354.2003. 12933882 PMC187310

[jkag075-B6] Bader T et al 2006. Role of calcineurin in stress resistance, morphogenesis, and virulence of a *Candida albicans* wild-type strain. Infect Immun. 74:4366–4369. 10.1128/IAI.00142-06. 16790813 PMC1489686

[jkag075-B7] Barelle CJ et al 2004. GFP as a quantitative reporter of gene regulation in *Candida albicans*. Yeast. 21:333–340. 10.1002/yea.1099. 15042593

[jkag075-B8] Bedekovic T, Agnew E, Brand AC. 2020. Rsr1 palmitoylation and GTPase activity status differentially coordinate nuclear, septin, and vacuole dynamics in *Candida albicans*. mBio. 11:. 10.1128/mBio.01666-20. PMC7554666 33051364

[jkag075-B9] Blankenship JR et al 2003. Calcineurin is essential for *Candida albicans* survival in serum and virulence. Eukaryot Cell. 2:422–430. 10.1128/EC.2.3.422-430.2003. 12796287 PMC161442

[jkag075-B10] Blankenship JR, Heitman J. 2005. Calcineurin is required for *Candida albicans* to survive calcium stress in serum. Infect Immun. 73:5767–5774. 10.1128/IAI.73.9.5767-5774.2005. 16113294 PMC1231066

[jkag075-B11] Brand A, Maccallum DM, Brown AJP, Gow NAR, Odds FC. 2004. Ectopic expression of *URA3* can influence the virulence phenotypes and proteome of *Candida albicans* but can be overcome by targeted reintegration of *URA3* at the *RPS10* locus. Eukaryot Cell. 3:900–909. 10.1128/EC.3.4.900-909.2004. 15302823 PMC500875

[jkag075-B12] Brand A et al 2007. Hyphal orientation of *Candida albicans* is regulated by a calcium-dependent mechanism. Curr Biol. 17:347–352. 10.1016/j.cub.2006.12.043. 17275302 PMC1885950

[jkag075-B13] Cao C, Cao Z, Yu P, Zhao Y. 2020. Genome-wide identification for genes involved in sodium dodecyl sulfate toxicity in *Saccharomyces cerevisiae*. BMC Microbiol. 20:34. 10.1186/s12866-020-1721-2. 32066383 PMC7027087

[jkag075-B14] Carmona-Gutierrez D et al 2018. Guidelines and recommendations on yeast cell death nomenclature. Microb Cell. 5:4–31. 10.15698/mic2018.01.607. 29354647 PMC5772036

[jkag075-B15] Carolus H, Pierson S, Lagrou K, Van Dijck P. 2020. Amphotericin B and other polyenes—discovery, clinical use, mode of action and drug resistance. J Fungi. 6:321. 10.3390/jof6040321. PMC7724567 33261213

[jkag075-B16] Chandrasekar P . 2011. Management of invasive fungal infections: a role for polyenes. J Antimicrob Chemother. 66:457–465. 10.1093/jac/dkq479. 21172787

[jkag075-B17] Chen S-Y, Chang C-K, Lan C-Y. 2023. Antimicrobial peptide LL-37 disrupts plasma membrane and calcium homeostasis in *Candida albicans* via the Rim101 pathway. Microbiol Spectr. 11:e0255123. 10.1128/spectrum.02551-23. 37888991 PMC10715129

[jkag075-B18] Chen YL et al 2011. Calcineurin controls drug tolerance, hyphal growth, and virulence in *Candida dubliniensis*. Eukaryot Cell. 10:803–819. 10.1128/EC.00310-10. 21531874 PMC3127677

[jkag075-B19] Cormack BP et al 1997. Yeast-enhanced green fluorescent protein (yEGFP): a reporter of gene expression in *Candida albicans*. Microbiology. 143:303–311. 10.1099/00221287-143-2-303. 9043107

[jkag075-B20] Cornish J, Chamberlain SG, Owen D, Mott HR. 2020. Intrinsically disordered proteins and membranes: a marriage of convenience for cell signalling? Biochem Soc Trans. 48:2669–2689. 10.1042/BST20200467. 33155649 PMC7752083

[jkag075-B21] Corvest V, Bogliolo S, Follette P, Arkowitz RA, Bassilana M. 2013. Spatiotemporal regulation of Rho1 and Cdc42 activity during *Candida albicans* filamentous growth. Mol Microbiol. 89:626–648. 10.1111/mmi.12302. 23796158

[jkag075-B22] Curtis A, Binder U, Kavanagh K. 2022. *Galleria mellonella* larvae as a model for investigating fungal—host interactions. Front Fungal Biol. 3:893494. doi:10.3389/ffunb.2022.893494. 37746216 PMC10512315

[jkag075-B23] De Backer MD et al 1999. Transformation of *Candida albicans* by electroporation. Yeast. 15:1609–1618. 10.1002/(sici)1097-0061(199911)15:15<1609::aid-yea485>3.3.co;2-p. 10572258

[jkag075-B24] Dennison PMJ, Ramsdale M, Manson CL, Brown AJP. 2005. Gene disruption in *Candida albicans* using a synthetic, codon-optimised Cre-loxP system. Fungal Genet Biol. 42:737–748. 10.1016/j.fgb.2005.05.006. 16043373

[jkag075-B25] Douglas LM, Konopka JB. 2016. Plasma membrane organization promotes virulence of the human fungal pathogen *Candida albicans*. J Microbiol. 54:178–191. 10.1007/s12275-016-5621-y. 26920878 PMC5650914

[jkag075-B100] Fonzi WA, Irwin MY. 1993. Isogenic strain construction and gene mapping in Candida albicans. Genetics 134, 717–728. 10.1093/genetics/134.3.717. 8349105 PMC1205510

[jkag075-B101] Gillum AM, Tsay EY Kirsch DR. 1984. Isolation of Candida albicans gene for orotidine-5′-phosphate decarboxylase by complementation of *Saccharomyces cerevisiae ura3 and Saccharomyces cerevisiae ura3* mutations. Saccharomyces cerevisiae ura3 198, 179–182. 10.1007/BF00328721. 6394964

[jkag075-B26] Giuraniuc CV et al 2023. Dynamic calcium-mediated stress response and recovery signatures in the fungal pathogen, *Candida albicans*. mBio. 14:e0115723. 10.1128/mbio.01157-23. 37750683 PMC10653887

[jkag075-B27] Gow NAR, Latge J, Munro CA. 2017. The fungal cell wall : structure, biosynthesis, and function. Microbiol Spectr. 5:1–25. 10.1128/microbiolspec.FUNK-0035-2016. PMC11687499 28513415

[jkag075-B28] Harpf V, Rambach G, Würzner R, Lass-Flörl C, Speth C. 2020. Candida and complement: new aspects in an old battle. Front Immunol. 11:1471. 10.3389/fimmu.2020.01471. 32765510 PMC7381207

[jkag075-B29] Jimenez AJ et al 2014. ESCRT machinery is required for plasma membrane repair. Science. 343:1247136. 10.1126/science.1247136. 24482116

[jkag075-B30] Jimenez AJ, Perez F. 2017. Plasma membrane repair: the adaptable cell life-insurance. Curr Opin Cell Biol. 47:99–107. 10.1016/j.ceb.2017.03.011. 28511145

[jkag075-B31] Karababa M et al 2006. *CRZ1*, a target of the calcineurin pathway in *Candida albicans*. Mol Microbiol. 59:1429–1451. 10.1111/j.1365-2958.2005.05037.x. 16468987

[jkag075-B32] Kitaura Y, Matsumoto S, Satoh H, Hitomi K, Maki M. 2001. Peflin and ALG-2, members of the penta-EF-hand protein family, form a heterodimer that dissociates in a Ca^2+^-dependent manner. J Biol Chem. 276:14053–14058. 10.1074/jbc.M008649200. 11278427

[jkag075-B33] Klis FM, Mol P, Hellingwerf K, Brul S. 2002. Dynamics of cell wall structure in *Saccharomyces cerevisiae*. FEMS Microbiol Rev. 26:239–256. 10.1111/j.1574-6976.2002.tb00613.x. 12165426

[jkag075-B34] Köhler JR, Hube B, Puccia R, Casadevall A, Perfect JR. 2017. Fungi that infect humans. Microbiol Spectr. 5:10.1128/microbiolspec.funk-0014–2016. 10.1128/microbiolspec.FUNK-0014-2016. PMC11687496 28597822

[jkag075-B35] Kumamoto CA, Gresnigt MS, Hube B. 2020. The gut, the bad and the harmless: *Candida albicans* as a commensal and opportunistic pathogen in the intestine. Curr Opin Microbiol. 56:7–15. 10.1016/j.mib.2020.05.006. 32604030 PMC7744392

[jkag075-B36] la Cour JM et al 2018. ALG-2 participates in recovery of cells after plasma membrane damage by electroporation and digitonin treatment. PLoS One. 13:e0204520. 10.1371/journal.pone.0204520. 30240438 PMC6150531

[jkag075-B37] Lanze CE et al 2020. Plasma membrane MCC/eisosome domains promote stress resistance in fungi. Microbiol Mol Biol Rev. 84:1–29. 10.1128/MMBR.00063-19. PMC7498080 32938742

[jkag075-B38] Lapaquette P et al 2022. Membrane protective role of autophagic machinery during infection of epithelial cells by *Candida albicans*. Gut Microbes. 14:2004798. 10.1080/19490976.2021.2004798. 35086419 PMC8803057

[jkag075-B39] Lee Y, Puumala E, Robbins N, Cowen LE. 2021. Antifungal drug resistance: molecular mechanisms in *Candida albicans* and beyond. Chem Rev. 121:3390–3411. 10.1021/acs.chemrev.0c00199. 32441527 PMC8519031

[jkag075-B40] Maki M, Kitaura Y, Satoh H, Ohkouchi S, Shibata H. 2002. Structures, functions and molecular evolution of the penta-EF-hand Ca^2+^-binding proteins. Biochim Biophys Acta BBA—Proteins Proteomics. 1600:51–60. 10.1016/S1570-9639(02)00444-2. 12445459

[jkag075-B41] Moyes DL et al 2016. Candidalysin is a fungal peptide toxin critical for mucosal infection. Nature. 532:64–68. 10.1038/nature17625. 27027296 PMC4851236

[jkag075-B42] Muñoz A et al 2020. Live-cell imaging of rapid calcium dynamics using fluorescent, genetically-encoded GCaMP probes with *Aspergillus fumigatus*. Fungal Genet Biol. 151:103470. 10.1016/j.fgb.2020.103470. 32979514 PMC7617832

[jkag075-B43] Murad AMA, Lee PR, Broadbent IAND, Barelle CJ, Brown AJP. 2000. CIp10, an efficient and convenient integrating vector for *Candida albicans*. Yeast. 8:1999–2001. 10.1002/1097-0061(20000315)16:4<325::AID-YEA538>3.0.CO;2-#. 10669870

[jkag075-B44] Noble SM, Gianetti BA, Witchley JN. 2017. *Candida albicans* cell-type switching and functional plasticity in the mammalian host—supporting information. Nat Rev Microbiol. 15:96–108. 10.1038/nrmicro.2016.157. 27867199 PMC5957277

[jkag075-B45] Noble SM, Johnson AD. 2005. Strains and strategies for large-scale gene deletion studies of the diploid human fungal pathogen *Candida albicans*. Eukaryot Cell. 4:298–309. 10.1128/EC.4.2.298-309.2005. 15701792 PMC549318

[jkag075-B46] Onyewu C, Wormley FL, Perfect JR, Heitman J. 2004. The calcineurin target, Crz1, functions in azole tolerance but is not required for virulence of *Candida albicans*. Infect Immun. 72:7330–7333. 10.1128/IAI.72.12.7330-7333.2004. 15557662 PMC529171

[jkag075-B47] Park H-S, Lee SC, Cardenas ME, Heitman J. 2019. Calcium-calmodulin-calcineurin signaling: a globally conserved virulence cascade in eukaryotic microbial pathogens. Cell Host Microbe. 26:453–462. 10.1016/j.chom.2019.08.004. 31600499 PMC6788756

[jkag075-B48] Perfect JR . 2017. The antifungal pipeline: a reality check. Nat Rev Drug Discov. 16:603–616. 10.1038/nrd.2017.46. 28496146 PMC5760994

[jkag075-B49] Perlin DS, Rautemaa-Richardson R, Alastruey-Izquierdo A. 2017. The global problem of antifungal resistance: prevalence, mechanisms, and management. Lancet Infect Dis. 17:e383–e392. 10.1016/S1473-3099(17)30316-X. 28774698

[jkag075-B50] Puumala E, Fallah S, Robbins N, Cowen LE. 2024. Advancements and challenges in antifungal therapeutic development. Clin Microbiol Rev. 37:e00142-23. 10.1128/cmr.00142-23. 38294218 PMC10938895

[jkag075-B51] Reedy JL, Filler SG, Heitman J. 2010. Elucidating the *Candida albicans* calcineurin signaling cascade controlling stress response and virulence. Fungal Genet Biol. 47:107–116. 10.1016/j.fgb.2009.09.002. 19755168 PMC2815136

[jkag075-B52] Sakaguchi S, Shibuya K, Lida H, Anraku Y, Suzuki T. 1997. Roles of Ca^2+^ in hyphal and yeast-form growth in *Candida albicans*. Growth regulation by altered extracellular and intracellular free Ca^2+^ concentrations. Mycoscience. 38:215–225. 10.1007/BF02460856.

[jkag075-B53] Sanglard D, Ischer F, Marchetti O, Entenza J, Bille J. 2003. Calcineurin A of *Candida albicans*: involvement in antifungal tolerance, cell morphogenesis and virulence. Mol Microbiol. 48:959–976. 10.1046/j.1365-2958.2003.03495.x. 12753189

[jkag075-B54] Sargeant J et al 2021. ALG-2 and peflin regulate COPII targeting and secretion in response to calcium signaling. J Biol Chem. 297:101393. 10.1016/j.jbc.2021.101393. 34762908 PMC8671942

[jkag075-B55] Scheffer LL et al 2014. Mechanism of Ca^2+^-triggered ESCRT assembly and regulation of cell membrane repair. Nat Commun. 5:5646. 10.1038/ncomms6646. 25534348 PMC4333728

[jkag075-B56] Schumann MR, Brandt U, Adis C, Hartung L, Fleißner A. 2019. Plasma membrane integrity during cell-cell fusion and in response to pore-forming drugs is promoted by the penta-EF-hand protein PEF1 in *Neurospora crassa*. Genetics. 213:195–211. 10.1534/genetics.119.302363. 31270133 PMC6727798

[jkag075-B57] Serrano A, Basante-Bedoya MA, Bassilana M, Arkowitz RA. 2023. A live-cell ergosterol reporter for visualization of the effects of fluconazole on the human fungal pathogen *Candida albicans*. mBio.e02493-23. 10.1128/mbio.02493-23. 38032182 PMC10746211

[jkag075-B58] Shahana S et al 2014. New clox systems for rapid and efficient gene disruption in *Candida albicans*. PLoS One. 9:e100390. 10.1371/journal.pone.0100390. 24940603 PMC4062495

[jkag075-B59] Sheehan G, Kavanagh K. 2019. Proteomic analysis of the responses of *Candida albicans* during infection of *Galleria mellonella* larvae. J Fungi. 5:7. 10.3390/jof5010007. PMC6463115 30641883

[jkag075-B60] Shen J, Guo W, Köhler JR. 2005. CaNAT1, a heterologous dominant selectable marker for transformation of *Candida albicans* and other pathogenic *Candida* species. Infect Immun. 73:1239–1242. 10.1128/IAI.73.2.1239-1242.2005. 15664973 PMC547112

[jkag075-B61] Shukla S et al 2024. Mechanism and cellular function of direct membrane binding by the ESCRT and ERES-associated Ca^2+^ -sensor ALG-2. Proc Natl Acad Sci U S A. 121:e2318046121. 10.1073/pnas.2318046121. 38386713 PMC10907313

[jkag075-B62] Struyfs C, Cammue BPA, Thevissen K. 2021. Membrane-interacting antifungal peptides. Front Cell Dev Biol. 9:649875. 10.3389/fcell.2021.649875. 33912564 PMC8074791

[jkag075-B63] Sudbery PE . 2011. Growth of *Candida albicans* hyphae. Nat Rev Microbiol. 9:737–748. 10.1038/nrmicro2636. 21844880

[jkag075-B64] Takeshita N, Higashitsuji Y, Konzack S, Fischer R. 2008. Apical sterol-rich membranes are essential for localizing cell end markers that determine growth directionality in the filamentous fungus *Aspergillus nidulans*. Mol Biol Cell. 19:339–351. 10.1091/mbc.e07-06-0523. 18003978 PMC2174190

[jkag075-B65] Thompson JR, Register E, Curotto J, Kurtz M, Kelly R. 1998. An improved protocol for the preparation of yeast cells for transformation by electroporation. Yeast. 14:565–571. 10.1002/(SICI)1097-0061(19980430)14:6<565::AID-YEA251>3.0.CO;2-B. 9605506

[jkag075-B66] Trdá L et al 2019. Dual mode of the saponin aescin in plant protection: antifungal agent and plant defense elicitor. Front Plant Sci. 10:1448. 10.3389/fpls.2019.01448. 31850004 PMC6893899

[jkag075-B67] Tsai CJ-Y, Loh JMS, Proft T. 2016. *Galleria mellonella* infection models for the study of bacterial diseases and for antimicrobial drug testing. Virulence. 7:214–229. 10.1080/21505594.2015.1135289. 26730990 PMC4871635

[jkag075-B68] Vernarecci S et al 2007. The yeast penta-EF protein Pef1p is involved in cation-dependent budding and cell polarization. Mol Microbiol. 65:1122–1138. 10.1111/j.1365-2958.2007.05852.x. 17640275

[jkag075-B69] Weichert M et al 2020. Plasma membrane fusion is specifically impacted by the molecular structure of membrane sterols during vegetative development of *Neurospora crassa*. Genetics. 216:1103–1116. 10.1534/genetics.120.303623. 33046504 PMC7768248

[jkag075-B70] Westman J, Hube B, Fairn GD. 2019. Integrity under stress: host membrane remodelling and damage by fungal pathogens. Cell Microbiol. 21:e13016. 10.1111/cmi.13016. 30740852

[jkag075-B71] Westman J, Moran G, Mogavero S, Hube B, Grinstein S. 2018. *Candida albicans* hyphal expansion causes phagosomal membrane damage and luminal alkalinization. mBio. 9:1–14. 10.1128/mBio.01226-18. PMC6134096 30206168

[jkag075-B72] Westman J et al 2022. Calcium-dependent ESCRT recruitment and lysosome exocytosis maintain epithelial integrity during *Candida albicans* invasion. Cell Rep. 38:110187. 10.1016/j.celrep.2021.110187. 34986345 PMC8755444

[jkag075-B73] Yadav V, Heitman J. 2023. Calcineurin: the achilles’ heel of fungal pathogens. PLOS Pathog. 19:e1011445. 10.1371/journal.ppat.1011445. 37410706 PMC10325075

[jkag075-B74] You Y, van Kan JAL. 2021. Bitter and sweet make tomato hard to (b)eat. New Phytol. 230:90–100. 10.1111/nph.17104. 33220068 PMC8126962

[jkag075-B75] You Y et al 2024. *Botrytis cinerea* combines four molecular strategies to tolerate membrane-permeating plant compounds and to increase virulence. Nat Commun. 15:6448. 10.1038/s41467-024-50748-5. 39085234 PMC11291775

[jkag075-B76] Zheng XD, Wang YM, Wang Y. 2003. *CaSPA2* is important for polarity establishment and maintenance in *Candida albicans*. Mol Microbiol. 49:1391–1405. 10.1046/j.1365-2958.2003.03646.x. 12940995

