# Supplementary material for: The penta-EF-hand protein Pef1 of Candida albicans functions at sites of membrane perturbation to support polarized growth and membrane integrity
Source: G3 (Bethesda). 2026 Apr 1;16(6):jkag075. doi: 10.1093/g3journal/jkag075 (PMC13232526; doi:10.1093/g3journal/jkag075)
Supplement: jkag075_Supplementary_Data [file jkag075_supplementary_data.zip › Figure_S1_G3-2026-406655.pdf]

A

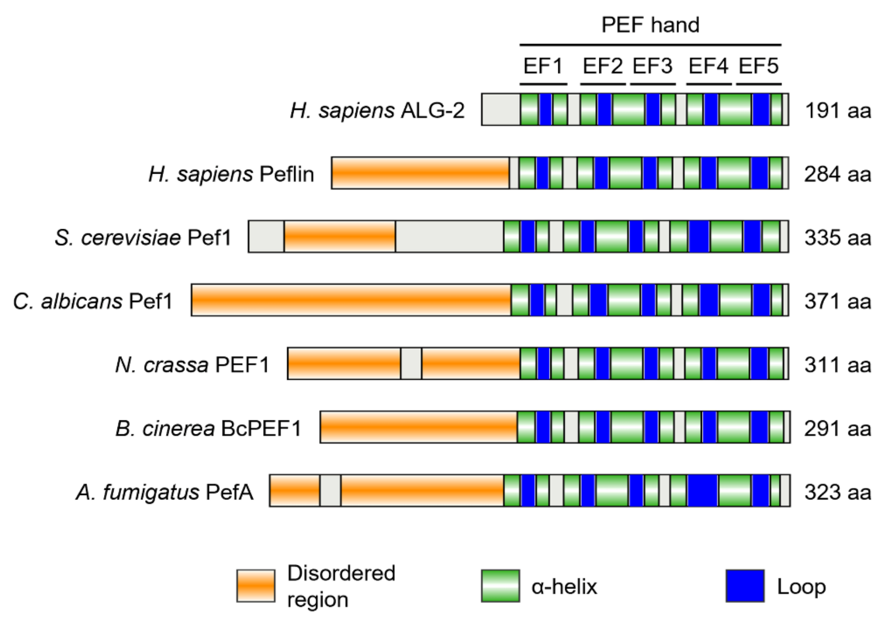

B

Disordered regions (Similarity [%] ↓ / Identity [%] →)	*Hs* ALG2	*Hs* Peflin	*Sc* Pef1	*Ca* Pef1	*Nc* PEF1	*Bc* PEF1	*Af* PefA
Hs ALG2	-	n/a	n/a	n/a	n/a	n/a	n/a
Hs Peflin	n/a	-	10.61	16.91	12.14	15.63	15.00
Sc Pef1	n/a	16.76	-	14.66	12.89	14.79	18.78
Ca Pef1	n/a	18.84	21.55	-	16.59	15.42	14.29
Nc PEF1	n/a	14.45	19.07	22.58	-	31.68	21.47
Bc PEF1	n/a	20.00	24.26	18.22	37.89	-	30.92
Af PefA	n/a	18.33	24.31	19.05	27.12	37.50	-

PEF-hand regions (Similarity [%] ↓ / Identity [%] →)	*Hs* ALG2	*Hs* Peflin	*Sc* Pef1	*Ca* Pef1	*Nc* PEF1	*Bc* PEF1	*Af* PefA
Hs ALG2	-	40.83	23.73	31.21	42.26	44.64	36.52
Hs Peflin	59.17	-	22.47	33.14	40.48	39.88	35.96
Sc Pef1	48.02	44.38	-	30.60	29.94	27.12	25.13
Ca Pef1	51.45	49.71	54.10	-	37.36	36.21	33.70
Nc PEF1	61.90	57.14	47.46	55.17	-	76.65	62.36
Bc PEF1	63.69	55.95	44.63	55.75	83.83	-	60.67
Af PefA	58.99	54.49	43.32	53.80	75.28	72.47	-

Fig. S1: Alignment of mammalian and fungal PEF hand-proteins.

A: Schematic representation of functional regions in the amino acid (aa) sequences of the *Homo sapiens* (*Hs*) proteins, ALG-2 and peflin (UniProt [https://www.uniprot.org/] entries: O75340 and Q9UBV8), and the orthologous penta-EF (PEF)-hand proteins from selected fungal species (FungiDB [https://fungidb.org/fungidb/app] entries: *Saccharomyces cerevisiae* [*Sc*], YGR058W; *Candida albicans* [*Ca*], C2_08020C_A; *Neurospora crassa* [*Nc*], NCU02738; *Botrytis cinerea* [*Nc*], Bcin06g03400; *Aspergillus fumigatus* [*Af*], Afu3g08540). The position of the conserved five EF domains, each composed of two α-helices and one loop, was determined with the web server “PredictProtein” (https://predictprotein.org/). Disordered regions were predicted with the integrative protein classification web tool “InterPro” (https://www.ebi.ac.uk/interpro/). The protein schematics were illustrated with the “IBS 2.0” (https://ibs.renlab.org/#/home) online tool.

B: Percentages of identity and similarity in between the aa sequences of the disordered regions (left) and the PEF-hand regions (right) from the human and fungal proteins presented in panel A. The values were calculated from aa alignments of these regions using the default setting of the online tool “Ident and Sim” at the Sequence Manipulation Suite (https://www.bioinformatics.org/sms2/index.html). All aa alignments were generated with the T-Coffee web server (https://tcoffee.crg.eu/apps/tcoffee/do:regular). n/a: not applicable.
