# Supplementary material for: The penta-EF-hand protein Pef1 of Candida albicans functions at sites of membrane perturbation to support polarized growth and membrane integrity
Source: G3 (Bethesda). 2026 Apr 1;16(6):jkag075. doi: 10.1093/g3journal/jkag075 (PMC13232526; doi:10.1093/g3journal/jkag075)
Supplement: jkag075_Supplementary_Data [file jkag075_supplementary_data.zip › Figure_S2_G3-2026-406655.pdf]

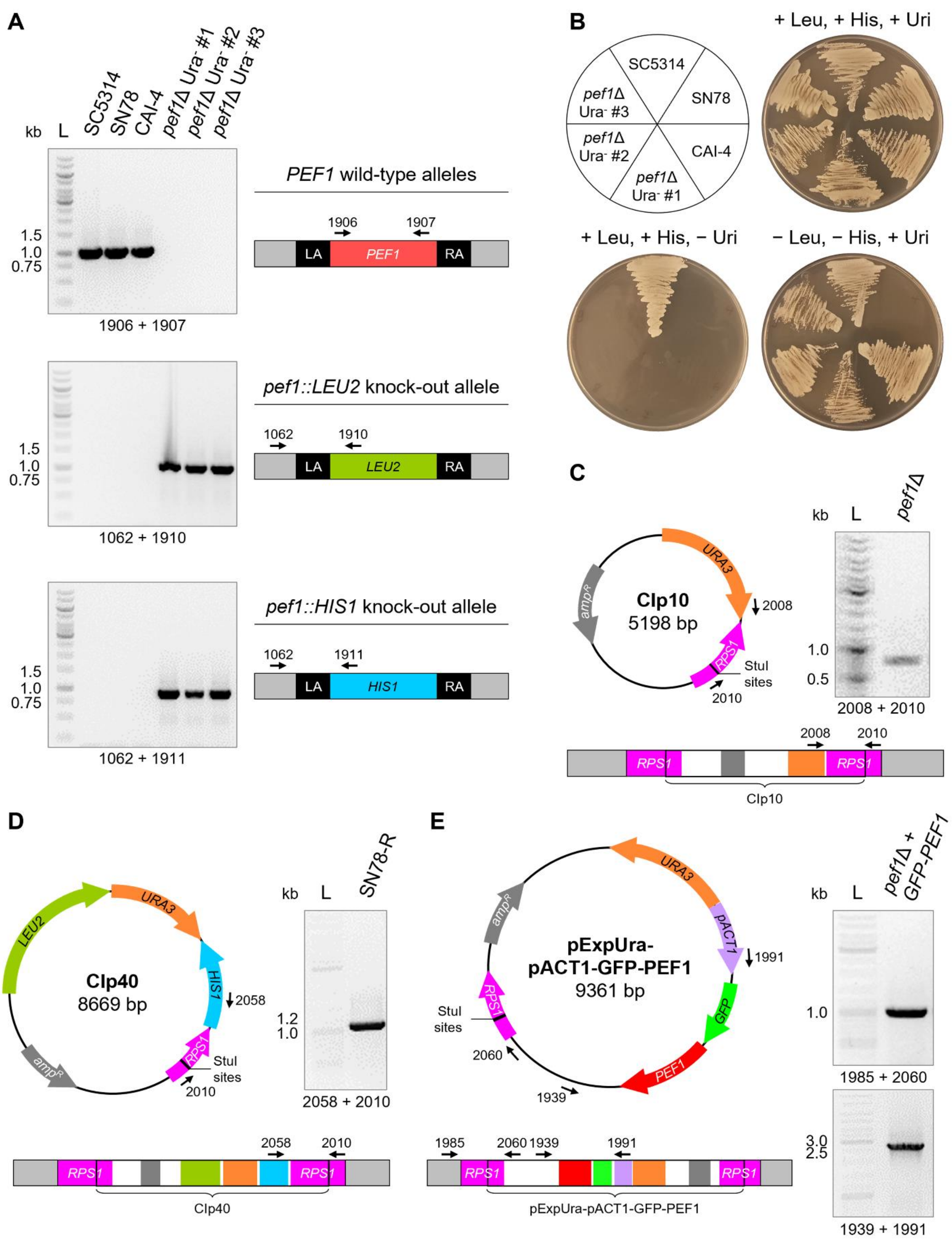

Fig. S2: Construction and verification of the *pef1*Δ mutant and the GFP-Pef1 reporter strain.

A: PCR analysis (left images) of three homozygous *pef1*Δ Ura$^-$ mutants (MS-01 to -03), their parental strain SN78, and two control strains (SC5314, CAI-4). The mutants were generated through successive replacement of both *PEF1* alleles in SN78 by gene

deletion cassettes containing the *LEU2* and *HIS1* selectable markers, respectively. The indicated primer pairs (numbered arrows) are specific for the wild-type or knock-out alleles (schematics to the right; not to scale).

B: Growth of the strains from panel A on plates containing SD medium with or without the indicated supplements (Leu: leucine, His: histidine, Uri: uridine). The images of the plates were captured after 2 d of incubation at 30°C.

C: PCR analysis (right image) of the prototrophic *pef1*Δ mutant (MW-Ca27) with the indicated primer pair to verify the integration of the *URA3* marker at the *RPS1* locus (linear schematic below) after transformation of the *pef1*Δ Ura$^-$ mutant (MS-01) with the StuI-linearized *Candida* integrating plasmid, CIp10 (circular schematic at the left).

D: PCR analysis of prototrophic parental strain, SN78-R (MW-Ca81), with the indicated primer pair. This control strain was generated through simultaneous re-integration of the *LEU2*, *URA3* and *HIS1* markers at the *RPS1* locus of SN78 via transformation with the StuI-linearized plasmid CIp40.

E: PCR analysis of the *pef1*Δ strain complemented with the *GFP-PEF1* construct (MW-Ca58) using the indicated primer pairs. Complementation of the *pef1*Δ Ura$^-$ mutant (MS-01) was achieved by transformation with the StuI-linearized vector pExpUra-pACT1-GFP-PEF1, which mediates constitutive expression of *GFP-PEF1* at the *RPS1* locus and also restores uracil prototrophy.
