# Supplementary material for: The penta-EF-hand protein Pef1 of Candida albicans functions at sites of membrane perturbation to support polarized growth and membrane integrity
Source: G3 (Bethesda). 2026 Apr 1;16(6):jkag075. doi: 10.1093/g3journal/jkag075 (PMC13232526; doi:10.1093/g3journal/jkag075)
Supplement: jkag075_Supplementary_Data [file jkag075_supplementary_data.zip › Figure_S3_G3-2026-406655.pdf]

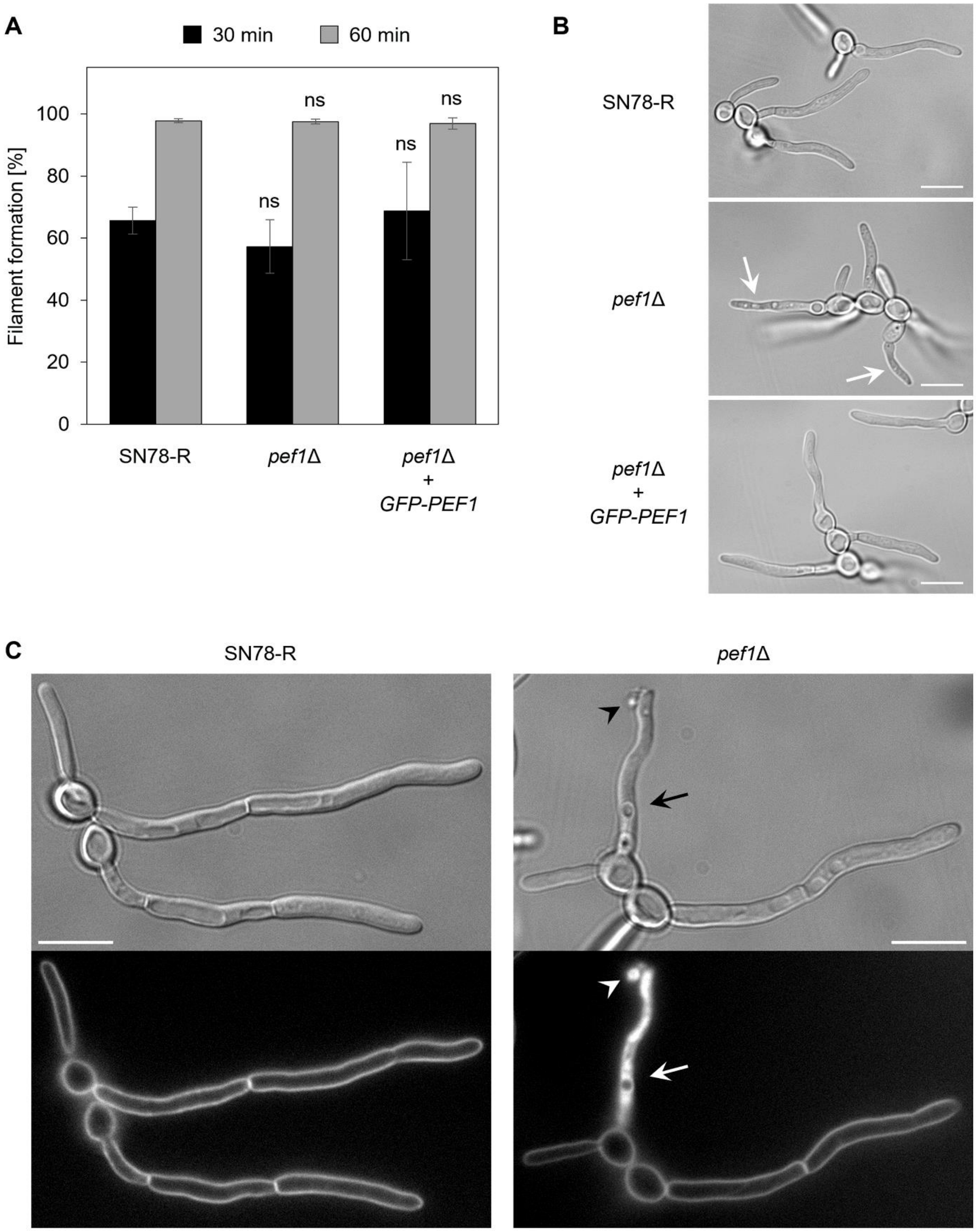

Fig. S3: Pef1 is dispensable for the induction of filamentation but required for maintaining hyphal integrity.

A: Quantification of the formation of filaments in the control strain SN78-R (MW-Ca81), the *pef1*Δ mutant (MW-Ca27) and the complemented mutant (MW-Ca58). Hyphae were induced by incubating yeast cells at 37°C in 20 % FBS. The percentage of filaments formed after 30 and 60 min was determined from three technical replicates

per strain and time point. Mean values (black and gray bars) and errors bars (Std Dev) were statistically compared with each other by one-way ANOVA analysis with Tukey's correction for multiple comparisons (ns, not significant).

B: Bright-field images of filaments of the strains from panel A after incubation for 2 h at 37°C in 20 % FBS. The hyphae of the *pef1*Δ mutant show signs of stress in the form of vacuolization (arrows). Scale bars: 10 µm.

C: FM4-64 staining of hyphae grown at 37°C in 20 % FBS. Fluorescence images (bottom) show evenly-distributed plasma membrane staining in the control filaments and some of the *pef1*Δ filaments. However, intense intracellular FM4-64 staining occurred, with some cell content leakage (small arrow), in some vacuolated *pef1*Δ hyphae (large arrow) similar to those shown in B. Bars: 10 µm.
