# Supplementary material for: The penta-EF-hand protein Pef1 of Candida albicans functions at sites of membrane perturbation to support polarized growth and membrane integrity
Source: G3 (Bethesda). 2026 Apr 1;16(6):jkag075. doi: 10.1093/g3journal/jkag075 (PMC13232526; doi:10.1093/g3journal/jkag075)
Supplement: jkag075_Supplementary_Data [file jkag075_supplementary_data.zip › Figure_S4_G3-2026-406655.pdf]

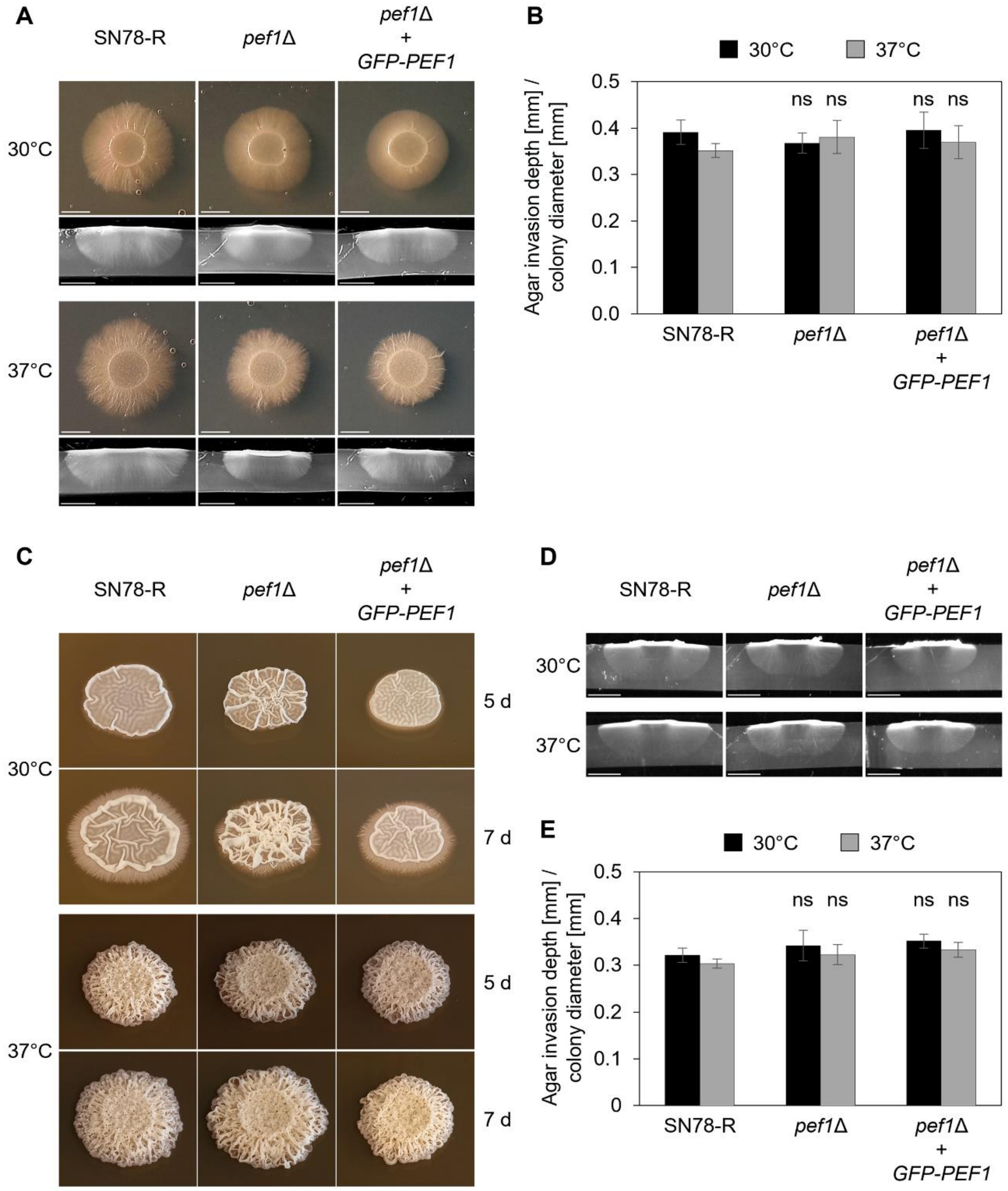

Fig. S4: Loss of Pef1 does not impair invasive filamentous growth of *C. albicans* on solid media.

A: Growth of SN78-R (MW-Ca81), the *pef1*Δ mutant (MW-Ca27) and the complemented strain (MW-Ca58) on solid serum medium (10 % FBS, 2 % agar) at 30°C and 37°C. The top images show the filamentation of the colonies after 7 d of incubation. Representative cross-sections prepared from the colonies are shown in the images below. Scale bars: 5 mm.

B: Quantification of the ratio between the average depth of agar invasion and the average diameter of colonies grown on serum agar as shown in panel A. Mean values and error bars (Std Dev) derived from two technical replicates were statistically

analyzed by one-way ANOVA analysis with Tukey’s correction for multiple comparisons (ns, not significant).

C: Representative colonies of the same strains as presented in panel A grown on solid Spider medium after 5 d and 7 d of incubation at 30°C and 37°C.

D: Images of representative cross-sections prepared from colonies grown on Spider agar as shown in panel C after 7 d of incubation at 30°C and 37°C. Scale bars: 5 mm.

E: Quantification of the ratio between the average depth of agar invasion and the average diameter of colonies grown on Spider agar as shown in C and D. Mean values and error bars (Std Dev) derived from four technical replicates were statistically analyzed by one-way ANOVA analysis with Tukey’s correction for multiple comparisons (ns, not significant).
