# Supplementary material for: The penta-EF-hand protein Pef1 of Candida albicans functions at sites of membrane perturbation to support polarized growth and membrane integrity
Source: G3 (Bethesda). 2026 Apr 1;16(6):jkag075. doi: 10.1093/g3journal/jkag075 (PMC13232526; doi:10.1093/g3journal/jkag075)
Supplement: jkag075_Supplementary_Data [file jkag075_supplementary_data.zip › Figure_S5_G3-2026-406655.pdf]

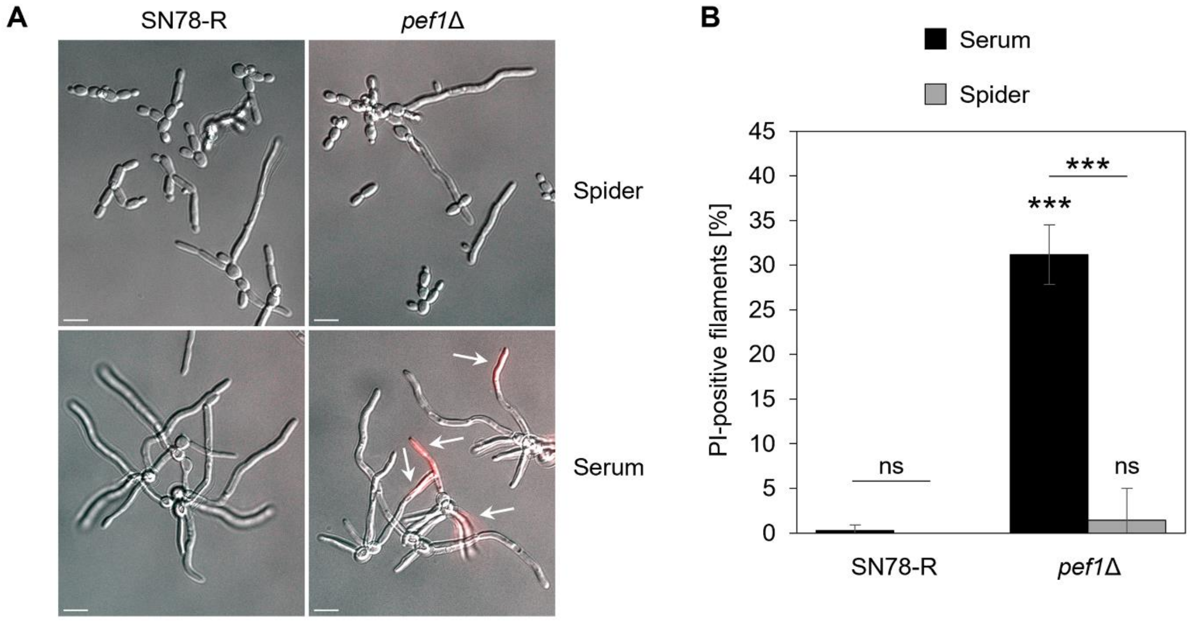

Fig. S5: Loss of Pef1 does not impair hyphal integrity in liquid Spider medium.

A: Filamentation of the *pef1*Δ mutant (MW-Ca27) and its control strain (MW-Ca81) after incubation at 37°C for 3-4 h in liquid Spider medium or 20 % FBS (serum). Images show overlays from DIC and fluorescence microscopy of cells stained with red-fluorescent propidium iodide (PI).

B: Quantification of the percentage of PI staining in filaments from the culture conditions shown in panel A. The black and gray bars represent mean values with errors (Std Dev) from 5-6 technical replicates obtained from two independent experiments per strain and medium. Statistically significant differences (***, $p < 0.001$; ns, not significant) were assessed by one-way ANOVA analysis with Tukey's correction for multiple comparisons
