# Supplementary material for: The penta-EF-hand protein Pef1 of Candida albicans functions at sites of membrane perturbation to support polarized growth and membrane integrity
Source: G3 (Bethesda). 2026 Apr 1;16(6):jkag075. doi: 10.1093/g3journal/jkag075 (PMC13232526; doi:10.1093/g3journal/jkag075)
Supplement: jkag075_Supplementary_Data [file jkag075_supplementary_data.zip › Figure_S6_G3-2026-406655.pdf]

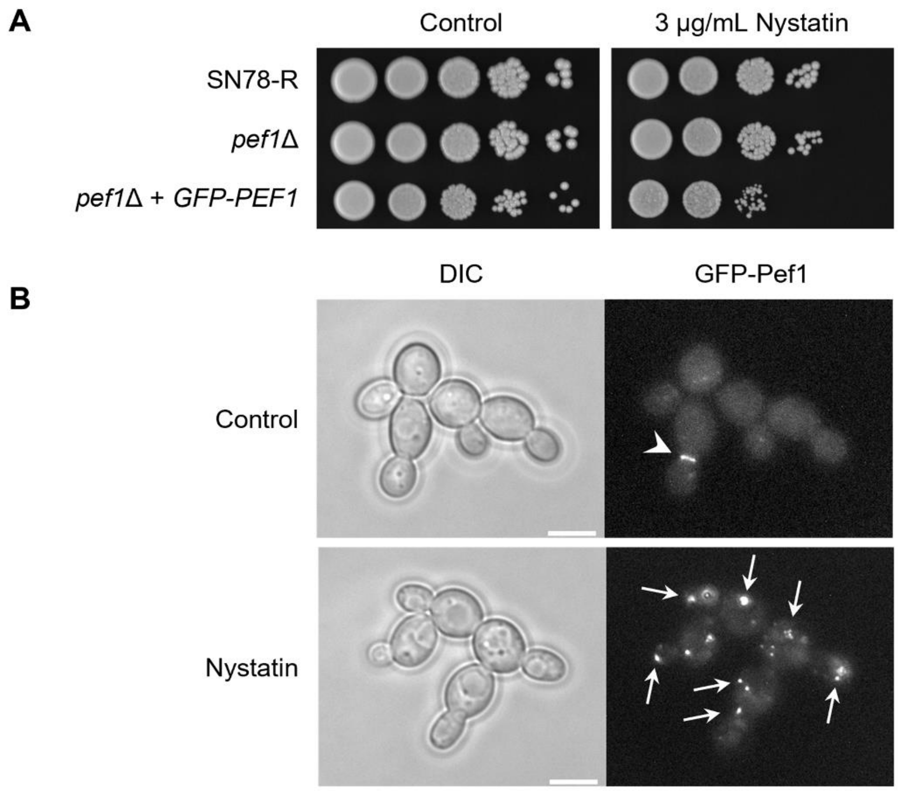

Fig. S6: Pef1 alters its localization pattern during the exposure of yeast cells to nystatin but is dispensable for the adaptation to this polyene.

A: Colonies grown from 10-fold serial dilutions of yeast cells of SN78-R (MW-Ca81), the *pef1*Δ mutant (MW-Ca27) and the complemented strain (MW-Ca58) were spotted on solid YPD medium with and without nystatin. The images of the plates were captured after incubation for 2 d at 37°C.

B: Localization of GFP-Pef1 in yeast cells of the reporter strain (MW-Ca58) exposed to nystatin. Cells from overnight cultures were grown for 6 h at 30°C in fresh YPD broth and washed in 0.9% saline prior to treatment with this polyene. Images were captured after about 10 min of incubation in the presence of 20 µg/mL nystatin or 0.2 % DMSO (control). Arrowhead: normal localization of GFP-Pef1 at the bud neck of dividing control cells; arrows: punctate accumulation of GFP-Pef1 at the cell periphery and at intracellular sites after antifungal treatment. Scale bars: 5 µm.
