# Supplementary material for: The penta-EF-hand protein Pef1 of Candida albicans functions at sites of membrane perturbation to support polarized growth and membrane integrity
Source: G3 (Bethesda). 2026 Apr 1;16(6):jkag075. doi: 10.1093/g3journal/jkag075 (PMC13232526; doi:10.1093/g3journal/jkag075)
Supplement: jkag075_Supplementary_Data [file jkag075_supplementary_data.zip › Table_S1_G3-2026-406655.pdf]

Table S1: List of primers used in this study

**Molecular cloning**		
**Number**	**Name**	**Nucleotide sequence (5’-3’)**
1050	Universal primer 2 (Noble & Johnson, 2005)	ccgctgctaggcgcgccgtgACCAGTGTGATGGATATCTGC
1051	Universal primer 5 (Noble & Johnson, 2005)	gcagggatgcggccgctgacAGCTCGGATCCACTAGTAACG
1052	PEF1 5´ flank-for	CCAGGGGTCGTGTATTTTTG
1053	PEF1 5´ flank-rev	cacggcgcgcctagcagcggGTTTATATAAAAAAAAAATTGG
1054	PEF1 3´ flank-for	gtcagcggccgcatccctgcAACGTGCAAGAGTTTTCAATG
1055	PEF1 3´ flank-rev	AGTTGGCACATTGACAGCAA
1953	CaURA3-SalI-SbfI-F	AATGTAGTCGACCTGCAGGCAATAATGCAGATTTGAAAACACCC
1954	CaURA3-XbaI-SpeI-R	AATGTATCTAGAACTAGTAGGACCACCTTTGATTGTAAATAG
1955	PEF1-RsrII-F1	AATGTACGGACCGtgATGGACGATTTGCCTCCTCAACCC
1956	PEF1-MluI-R	ATAGTAACGCGTTAGGTCTAGCAGCATTAGTACATGC
2024	CaLEU2-SacI-F	ATAGTAGAGCTCACACTGATGCTGCTCAGGCGTATGG
2025	CaLEU2-NotI-R	ATATTAGCGGCCGCGCCAAGTCAATCCGAATCAAGCG
MW-P1	CaNAT1-NotI-F	ATAGTAGCGGCCGCGATATCAAGCTTGCCTCGTCC
MW-P2	CaNAT1-SpeI-R	AATGTAACTAGTGTCGACACTGGATGGCGGCG
**Confirmatory PCR analysis**		
**Number**	**Name**	**Nucleotide sequence (5’-3’)**
1062	PEF1-test-for	GCCATTGACCAGATTGAAGAATGC
1906	PEF1-internal-F1	AACCCACACCTCAGCAATCCTACC
1907	PEF1-internal-R1	ATTGTGGCAGTACCAGACAAATCC
1910	CmLEU2-test-R	AAGTTGGTGACGCGATTGTCGAAGC
1911	CdHIS1-test-R	GGATGTTGTGTAAAATGCTGCGTAGC
1939	pGFP-RID-test-R	GCTATTTGCTTATTTACTGGTGTCC

1985	RPS1-test-F	CAACATCTCGTATTCACTTAATCCC
1991	pACT1-F	GCTGTTCTCACCAAGATTTATTGCC
2008	CaURA3-test-F	GGTATAGAAATGCTGGTTGG
2010	RPS1-CIp10-R	AAGTTTGTTTGGCCGACTTACAAGG
2058	CaHIS1-test-R	GGCAAGTTTGTAGAAAGTGC
2060	pACT1-test-R	CTGATGCGGTATTTTCTCCTTACGC
